# Asynchronous Replication, Mono-Allelic Expression, and Long Range *Cis*-Effects of *ASAR6*

**DOI:** 10.1371/journal.pgen.1003423

**Published:** 2013-04-04

**Authors:** Nathan Donley, Eric P. Stoffregen, Leslie Smith, Christina Montagna, Mathew J. Thayer

**Affiliations:** 1 Department of Biochemistry and Molecular Biology, Oregon Health and Science University, Portland, Oregon United States of America; 2 Department of Cell and Developmental Biology, Oregon Health and Science University, Portland, Oregon, United States of America; 3 Departments of Genetics and Pathology, Albert Einstein College of Medicine, Bronx, New York, United States of America; 4 Knight Cancer Institute, Oregon Health and Science University, Portland, Oregon, United States of America; University of Pennsylvania, United States of America

## Abstract

Mammalian chromosomes initiate DNA replication at multiple sites along their length during each S phase following a temporal replication program. The majority of genes on homologous chromosomes replicate synchronously. However, mono-allelically expressed genes such as imprinted genes, allelically excluded genes, and genes on female X chromosomes replicate asynchronously. We have identified a *cis*-acting locus on human chromosome 6 that controls this replication-timing program. This locus encodes a large intergenic non-coding RNA gene named Asynchronous replication and Autosomal RNA on chromosome 6, or *ASAR6*. Disruption of *ASAR6* results in delayed replication, delayed mitotic chromosome condensation, and activation of the previously silent alleles of mono-allelic genes on chromosome 6. The ASAR6 gene resides within an ∼1.2 megabase domain of asynchronously replicating DNA that is coordinated with other random asynchronously replicating loci along chromosome 6. In contrast to other nearby mono-allelic genes, ASAR6 RNA is expressed from the later-replicating allele. ASAR6 RNA is synthesized by RNA Polymerase II, is not polyadenlyated, is restricted to the nucleus, and is subject to random mono-allelic expression. Disruption of *ASAR6* leads to the formation of bridged chromosomes, micronuclei, and structural instability of chromosome 6. Finally, ectopic integration of cloned genomic DNA containing *ASAR6* causes delayed replication of entire mouse chromosomes.

## Author Summary

Mammalian chromosomes are duplicated every cell cycle during a precise temporal DNA replication program. Thus, every chromosome contains regions that are replicated early and other regions that are replicated late during each S phase. Most of the genes, present in two copies on homologous chromosomes, replicate synchronously during each S phase. Exceptions to this rule are genes located on X chromosomes, genetically imprinted genes, and genes subject to allelic exclusion. Thus, all mono-allelically expressed genes are subject to asynchronous replication, where one allele replicates before the other. Perhaps the best-studied example of asynchronous replication in mammals occurs during X inactivation in female cells. A large non-coding RNA gene called XIST, located within the X inactivation center, controls the transcriptional silencing and late replication of the inactive X chromosome. We have identified a locus on human chromosome 6 that shares many characteristics with XIST. This chromosome 6 locus encodes a large intergenic non-coding RNA gene, *ASAR6*, which displays random mono-allelic expression, asynchronous replication, and controls the mono-allelic expression of other genes on chromosome 6. Our work supports a model in which all mammalian chromosomes contain similar *cis*-acting loci that function to ensure proper chromosome replication, mitotic condensation, mono-allelic expression, and stability of individual chromosomes.

## Introduction

Morphological differences between mitotic chromosomes residing within the same cell were first described in mammalian cells over forty years ago (reviewed in [1]). In addition, numerous reports have described an abnormal chromosomal phenotype affecting single or a few chromosomes in mitotic preparations from tumor-derived and other established cell lines. For example, Dr. Harald zur Hausen described an under-condensed appearance of individual chromosomes during mitosis in cell lines derived from 7 different leukemia patients [2]. The individual under-condensed chromosomes synthesized DNA after the normally condensed chromosomes had finished replication, indicating that the under-condensed chromosomes were extremely late replicating, with DNA synthesis extending into the G2 phase. These observations have been extended to include chromosomes present in numerous tumor-derived and permanent cell lines from several different mammalian species [3], [4], [5]. Furthermore, the under-condensed and late replicating phenotype present in tumor-derived cells occurred only on certain rearranged chromosomes and not on others, suggesting that the under-condensed and late replicating phenotype was associated with certain chromosomal rearrangements [5]. A detailed analysis of the replication-timing defect on under-condensed chromosomes indicated that they exhibit a delay in replication timing (DRT), which is characterized by a >2 hour delay in both the initiation and the completion of DNA synthesis along the entire length of the chromosome [5]. Chromosomes with DRT also display a delay in mitotic chromosome condensation (DMC), which is characterized by an under-condensed appearance during mitosis, delayed recruitment of Aurora B kinase, and a delay in the mitotic phosphorylation of serine 10 of histone H3 [5], [6]. The DRT/DMC phenotype was also detected on ∼5% of inter-chromosomal translocations induced by exposure to ionizing radiation (IR) [7]. Taken together, these observations indicate that DRT/DMC occurs on certain rearranged chromosomes and is a common phenotype in cancer cells and in cells exposed to IR.

We have developed a chromosome engineering system that allows for the systematic analysis of human chromosomes with DRT/DMC [6], [7], [8], [9]. This system relies on site-specific recombinases to generate precise chromosomal rearrangements. Using this system we previously identified four balanced translocations, each displaying DRT/DMC on one of the two derivative chromosomes [8]. Subsequently, we found that translocations or deletions at a discrete locus on human chromosome 6 result in DRT/DMC. The deletions that cause DRT/DMC on chromosome 6 disrupt a large intergenic non-coding RNA gene named ASynchronous replication and Autosomal RNA on chromosome 6, or *ASAR6* [9]. *ASAR6* displays random mono-allelic expression in immortalized cell lines, and as the name implies, displays asynchronous replication between alleles. In addition, the asynchronous replication of *ASAR6* is coordinated with other asynchronous loci on the long arm of chromosome 6. Thus, the early replicating chromosome for *ASAR6* is also the early replicating chromosome for several nearby mono-allelic genes, indicating that these genes display *cis*-coordinated asynchronous replication. However, the early replicating chromosome for *ASAR6* was on the same homolog as the later replicating alleles for other random asynchronously replicating loci located at a distance on the long arm of chromosome 6, indicating that the coordination of asynchronous replication with these other genes was in *trans* [9].

*ASAR6* shares many physical and functional similarities with the large non-coding RNA gene Xist, which is located within the X inactivation center [10], [11]. For example, *ASAR6* and *Xist* represent large non-coding RNA genes that display random mono-allelic expression, asynchronous replication, and they both control the expression of other mono-allelic genes in *cis* [9], [10]. In addition, deletion of the *Xist* gene from somatic cells isolated from adult mice results in a late replication phenotype that is similar to the DRT phenotype caused by disruption of *ASAR6* [9], [12], [13]. Thus, the chromosomal phenotypes associated with *ASAR6* disruption are remarkably similar to the phenotypes associated with disruption of *Xist* in adult somatic cells (see Table 1) [8], [9], [13].

**Table 1:** Phenotypes associated with gene disruption.

	Delayed replication	Loss of mono-allelic expression	Structural instability	Ref.
**ASAR6 (translocation)**	**+**	**+**	**+**	**1**
**ASAR6 (deletion)**	**+**	**+**	**+**	**2**
**Xist (deletion in adult)**	**+**	**+**	**+**	**3**

1. Breger et al 2005.

2. Stoffregen et al 2011.

3. Diaz-Perez et al 2006.

In this report we mapped the domain of asynchronous replication surrounding *ASAR6*, and found that it resides within an ∼1.2 mb domain containing five other protein coding and non-coding genes at 6q16.1. In contrast to other nearby mono-allelic genes, *ASAR6* is expressed from the later replicating allele, which is another characteristic shared with *Xist* [14]. We also found that the asynchronous replication of *ASAR6* is also coordinated with other asynchronously replicating loci on the short arm of chromosome 6, indicating that the coordination in replication timing extends across the centromere. In addition, we found that *ASAR6* RNA is transcribed by RNA Polymerase II, even though it is not spliced nor polyadenlyated. Furthermore, we found that chromosomes with a disruption of *ASAR6* appear as bridged chromosomes between daughter nuclei, are often found in micronuclei, and experience frequent secondary rearrangements, indicating that *ASAR6* functions to maintain the structural integrity of chromosome 6 via a *cis* acting mechanism. Finally, we found that ectopic integration of cloned genomic DNA containing *ASAR6* can cause delayed replication of entire mouse chromosomes.

## Results

### *ASAR6* resides within an ∼1.2 mb domain of asynchronous replication

**Figure 1:**
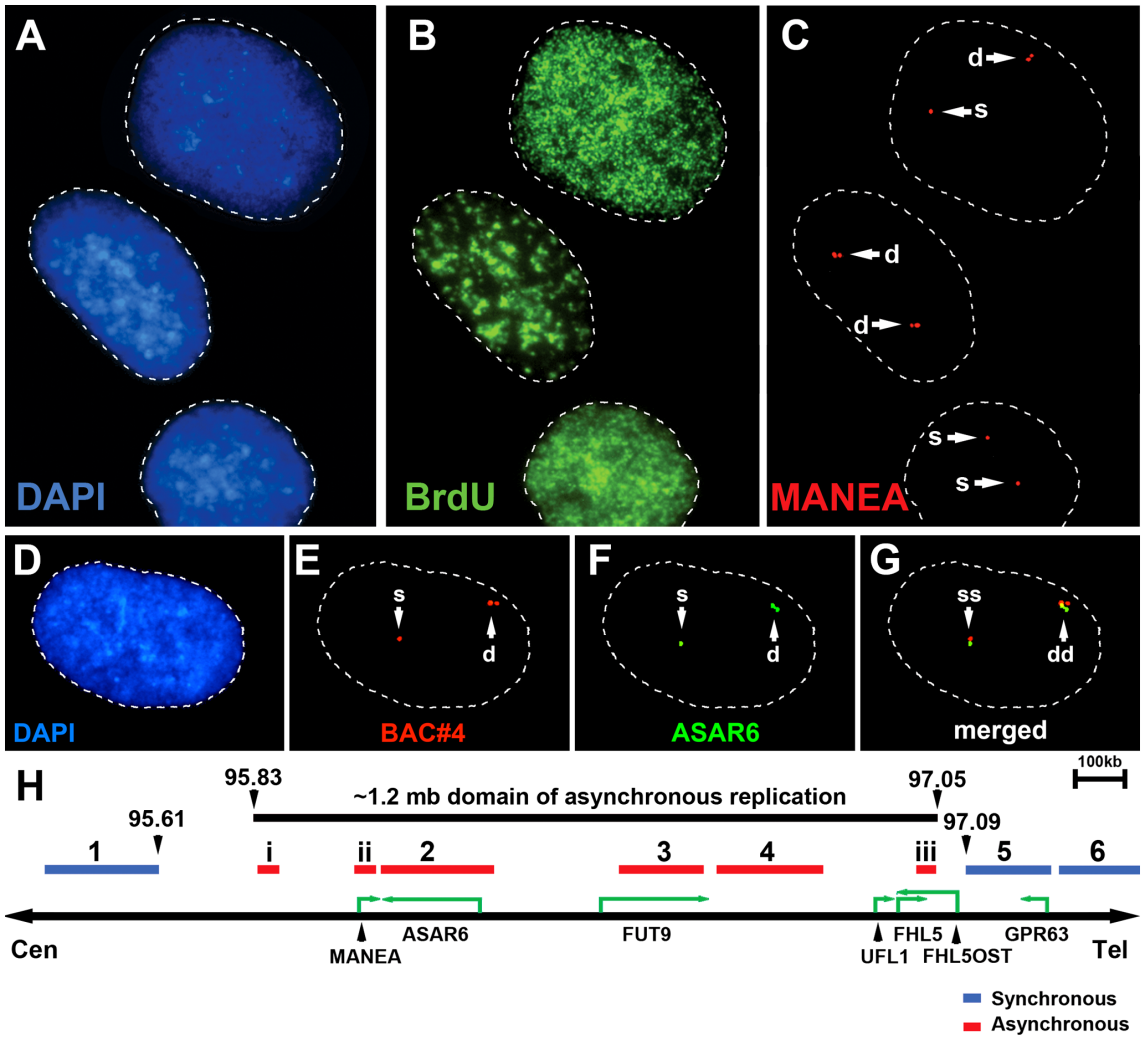
Asynchronous replication of the *ASAR6* domain. Low passage human skin fibroblasts were analyzed using the “single dot-double dot” assay [21]. A–C) Cells were processed for FISH using a Fosmid containing the *MANEA* gene (G248P8476B7) and for DNA synthesis using BrdU incorporation. A) DNA was stained with DAPI (blue). B) DNA synthesis was detected using an antibody against BrdU (green). C) DNA FISH was used to detect *MANEA* (red). Single dots (s) represent un-replicated loci and double dots (d) represent replicated loci. Approximately 35% of cells were BrdU positive. Arrows mark the location of the FISH signals. D–G) Two-color DNA FISH assay for coordination in asynchronous replication. Low passage primary human skin fibroblasts were processed for DNA FISH using BAC RP11-374I15 as probe (*ASAR6*, green in F and G) plus BAC RP11-959I6 (red in E and G, and *BAC#4* in H). The percentage of cells that simultaneously displayed the sd pattern for both probes (ss/dd) was determined in 100 cells (see Table 2). A–G) Dashed lines show the outline of the nuclei. DNA was stained with DAPI (blue). Arrows mark the location of the DNA FISH signals. H) Schematic diagram of the ∼1.2 mb region of chromosome 6 that displays *cis* coordination in asynchronous replication. The positions of the boundaries between synchronous and asynchronously replicating probes are shown in megabases. The probes that showed asynchronous replication are shown in red and the probes that showed synchronous replication are shown in blue. BAC RP11-922A18 (1 in panel H), BAC RP11-374I15 (2 in panel H) BAC RP11-75N19 (3 in panel H), BAC RP11-959I6 (4 in panel H), BAC CTD 2183011 (5 in panel H), BAC CTC-385F17 (6 in panel H), Fosmid G248P85637C5 (i in panel H), G248P8476B7 (ii in panel H), or G248P86054G4 (iii in panel H). Green arrows mark the location and direction of transcription of the indicated genes.

Mono-allelic expression with random choice between maternal and paternal alleles defines genes subject to X-inactivation and ∼5–10% of autosomal genes [10], [15], [16]. One defining characteristic of mono-allelically expressed genes is asynchronous replication timing between alleles [17], [18], [19], [20]. We previously found that *ASAR6* displays asynchronous replication in primary human fibroblasts, and that three genes located near *ASAR6* also display asynchronous replication [9]. In addition, the asynchronous replication of *ASAR6* and these nearby genes is coordinated in *cis*. However, the boundaries of this asynchronously replicating domain were not defined in this earlier study. Therefore, to characterize this *cis*-coordinated domain of asynchronous replication timing further, we examined the extent of asynchronous replication of loci extending centromeric and telomeric from *ASAR6*. For this analysis we used a “single dot-double dot” FISH assay [21] on primary human skin fibroblasts. This assay utilizes a methanol/acetic acid fixation, which destroys the nuclear structure and allows for a relatively accurate analysis of replication timing [19], [22]. Using a probe to a particular chromosomal site, some cells display two single hybridization dots, indicating that neither allele has replicated (a ss pattern), other cells display two double dots, indicating that both alleles have replicated (a dd pattern), and a third class of cells contains one single dot and one double dot (a sd pattern), indicating that only one of the two alleles has replicated (see Figure 1A–1C). For the analysis of the *ASAR6* domain we used two-color FISH to examine two loci simultaneously and scored cells that presented the sd pattern for both loci. If the two loci show asynchronous replication that is coordinated in *cis* the double dots for both loci will be on the same chromosome [[17]; see Figure 1D–1G]. In contrast, if the two loci are coordinated in *trans* the double dots will be on opposite chromosomes [9]. Furthermore, if the two loci are not coordinated, the double dots for both loci will be on the same chromosome 50% of the time. Table 2 shows the results of our analysis and indicates that *ASAR6* resides within an ∼1.2 megabase domain of *cis*-coordinated asynchronous replication surrounded by synchronously replicating DNA. Figure 1H illustrates the positions of the probes used during this analysis and the extent of asynchronous and synchronous replication surrounding *ASAR6*.

**Table 2:** Coordinated asynchronous replication timing by single-double assay.

		Coordination with ASAR6 (%)		
Locus	Position (CHR6)	cis	trans	P value
KCNQ5*	73,584,039–73,732,886	26	74	<1×10−6
ME1*	83,995,494–84,160,235	14	86	<1×10−6
HTRE1*	87,540,947–87,706,640	24	76	<1×10−6
MAP3K7	91,349,552–91,497,736	55	45	0.19
MAP3K7	91,446,216–91,616,242	53	47	0.3
TSG1	95,010,206–95,171,821	46	54	0.24
BAC #1	95,452,857–95,613,708	55	45	0.18
Fosmid i	95,832,625–95,876,472	86	14	<1×10−6
MANEA/Fosmid ii*	96,038,240–96,075,864	78	22	<1×10−6
ASAR6*/BAC#2	96,061,931–96,237,378	X	X	X
FUT9/BAC#3*	96,502,279–96,663,444	78	22	<1×10−6
UFL1/BAC#4*	96,687,004–96,881,427	70	30	<1×10−4
FHL5/Fosmid iii*	97,016,622–97,055,738	74	26	<1×10−6
BAC#5	97099204–97238887	57	43	0.09
NDUFAF4/BAC#6	97,290,673–97,431,128	50	50	0.53
KLHL32	97,501,882–97,663,681	65	35	0.02
MIR2113	98,211,202–98,374,876	55	45	0.18
PREP	105,673,074–105,873,728	44	56	0.14
FRK*	116,212,652–116,385,506	26	74	<1×10−6

* Indicates that the data was from Stoffregen et al. 2011.

### Asynchronous replication on chromosome 6 is coordinated in *cis* and in *trans*

**Figure 2:**
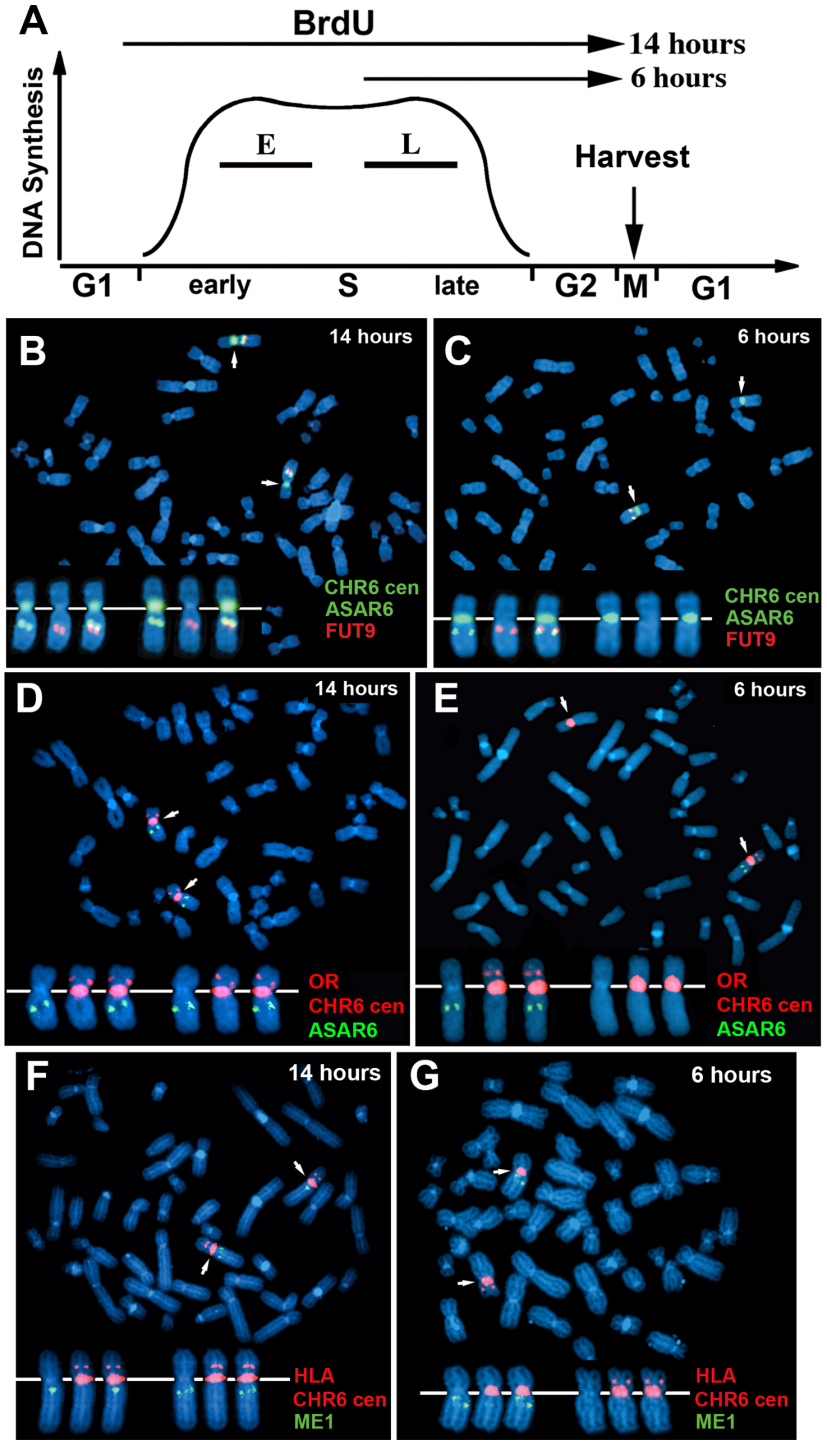
Coordinated asynchronous replication timing on chromosome 6. A) Schematic representation of the ReTiSH assay. PBLs were exposed to BrdU during the entire length of S phase (14 hours) or only during late S phase (6 hours). The ReTiSH assay can distinguish between alleles that replicate early (E) and late (L) in S phase. B–G) Mitotic spreads were hybridized with three different FISH probes. First, each ReTiSH assay included a centromeric probe to chromosome 6. Arrows mark the centromeric signals in each panel. Each assay also included BAC probes representing asynchronously replicating loci on chromosome 6. The insets in each panel show the two chromosome 6 s aligned at their centromeres. B and C) ReTiSH assay using an *ASAR6* BAC (RP11-374I15; green) plus a *FUT9* BAC (RP11-75N19; red). The *ASAR6* and *FUT9* BACs show hybridization signals to the same chromosome 6 at the 6 hour time point. D and E) ReTiSH assay using an *ASAR6* BAC (RP11-374I15; green) plus a BAC (RP11-150A6; red) containing the olfactory receptor genes OR14J1, OR5V1, OR12D3 and OR12D2. The *ASAR6* BAC and the OR BAC show hybridization signals to the same chromosome 6 s at the 6 hour time point. F and G) ReTiSH assay using an HLA BAC (RP11-166F24; red) plus a *ME1* BAC (RP11-703G14; green). HLA and *ME1* show hybridization signals on the opposite chromosome 6 s at the 6 hour time point, indicating that *HLA* and *ME1* display *trans* coordination of asynchronous replication.

One limitation of the “single dot-double dot” assay is that the asynchronous replication of loci greater than 50 mb apart are difficult to score, as a signal coming from the paternal allele of one locus may be closer to the maternal allele of the other locus. Therefore, to assay the random asynchronous replication of chromosome 6 loci at the whole chromosome level we utilized a second replication-timing assay known as Replication Timing-Specific Hybridization, or ReTiSH [23]. In the ReTiSH assay, cells are labeled with BrdU for different times and then harvested during metaphase (see Figure 2A). Regions of chromosomes that incorporate BrdU are visualized by a modification of Chromosome Orientation-Fluorescence In Situ Hybridization (CO-FISH), where the replicated regions (BrdU-labeled) are converted to single stranded DNA and then hybridized directly with specific probes [24]. Since metaphase chromosomes are analyzed for hybridization signals located on the same chromosome in metaphase spreads, the physical distance between the two loci is not a limitation of the ReTiSH assay [23]. To verify this methodology in our hands, we analyzed the hybridization patterns of ribosomal RNA genes. The rDNA clusters are located on human chromosomes 13, 14, 15, 21 and 22, and are known to display asynchronous replication between alleles [23]. In addition, to assay asynchronous replication in a second human cell type we used primary blood lymphocytes (PBLs) for this analysis. PBLs were exposed to BrdU for either 14 or 6 hours. As expected, we detected hybridization of an 18S rDNA probe to all 10 chromosomes at the 14 hour time point, but hybridization of this rDNA probe to only 5 chromosomes, representing single copies each of chromosomes 13, 14, 15, 21, and 22, at the 6 hour time point (Figure S1). Therefore, this assay allows us to identify early and late replicating chromosomes with respect to asynchronously replicating loci. Next, we used a two-color hybridization scheme to simultaneously detect *ASAR6* and other asynchronous loci on chromosome 6. This assay also included a chromosome 6 centromeric probe to unambiguously identify both chromosome 6 s. Because centromeric heterochromatin is late replicating, centromeric probes hybridize to both copies of each chromosome at the 14 and 6 hour time points [23]. As observed with the “single dot-double dot” assay ([9]; and see Table 2 and Figure 1), we found that the asynchronous replication of *ASAR6* was coordinated in *cis* with the closely linked genes *FUT9* and *UFL1* (Figure 2B and 2C; and Table 3). We also found that the asynchronous replication of *ASAR6* was coordinated with the *ME1* locus; however the coordination was in *trans* (Table 3). Thus, the earlier replicating *ASAR6* allele is on the same chromosome as the later replicating *ME1* allele, which was also seen previously using the “single dot-double dot” assay in primary fibroblasts [9]. In addition, we found that the asynchronous replication of *ASAR6* was also coordinated in *cis* with two loci on the short arm of chromosome 6, the HLA locus and an olfactory receptor (OR) gene cluster (Figure 2D and 2E; and Table 3). To confirm the *trans* coordination along chromosome 6 using additional probes, we assayed the *ME1* and HLA loci simultaneously using ReTiSH. As expected we found that the later replicating *ME1* allele was on the same chromosome as the earlier replicating chromosome for the HLA locus, and vise versa (Figure 2F and 2G; and Table 3). These data indicate that human chromosome 6 contains loci that display asynchronous replication that is coordinated both in *cis* and in *trans*, and that some of these loci are separated by >87 megabases of genomic DNA and located on either side of the centromere (Figure S2).

**Table 3:** Coordinated asynchronous replication timing by ReTiSH.

PBLs				
Locus 1	Locus 2	cis (%)	trans (%)	P value
ASAR6	FUT9	80	20	<1×10−4
ASAR6	UFL1	76	24	<1×10−3
ASAR6	ME1	26	74	<1×10−3
ASAR6	HLA	87	13	<1×10−6
ASAR6	OR	86	14	<1×10−6
ME1	HLA	18	82	<1×10−5

P175				
Locus 1	Locus 2	cis (%)	trans (%)	P value
ASAR6	CHR6 cen*	88	12	<1×10−6
FUT9	CHR6 cen*	96	4	<1×10−6
KIAA0776	CHR6 cen*	90	10	<1×10−6
ME1	CHR6 cen*	4	96	<1×10−6
HTR1E	CHR6 cen*	0	100	<1×10−6
ME1	HTRE1	78	22	<1×10−4
Aprt Transgene	CHR6 cen*	100	0	<1×10−6

CHR6 cen* indicates the large centromere on one of the chromosome 6 s in P175 cells.

### The expressed allele of *ASAR6* replicates after the silent allele

**Figure 3:**
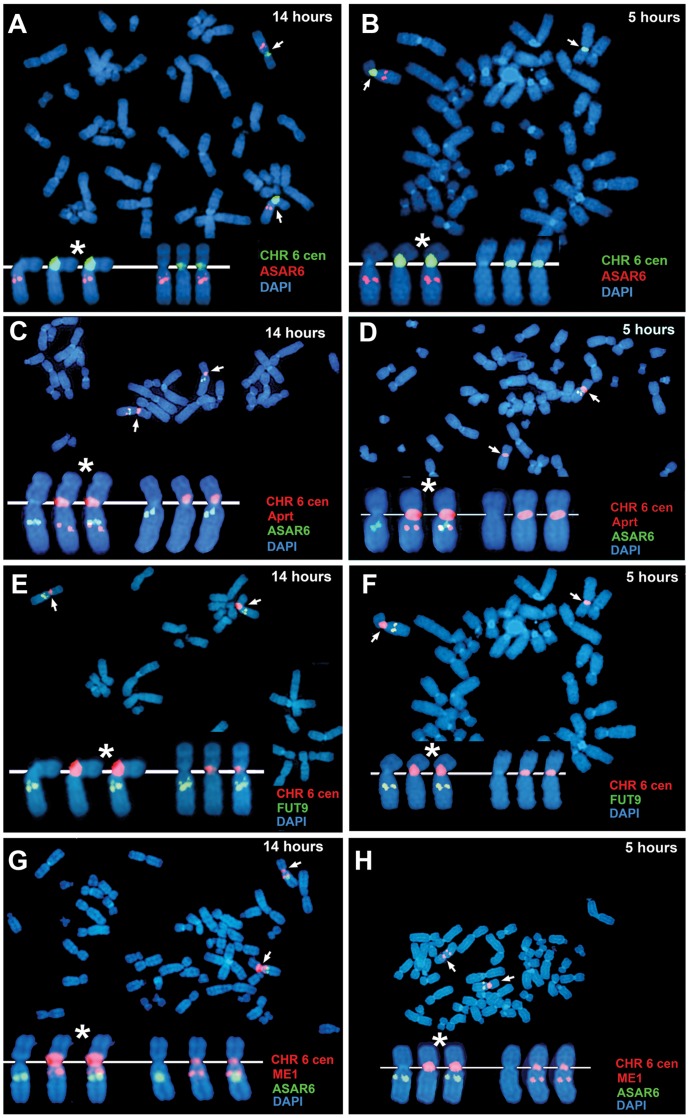
*ASAR6* is expressed from the later replicating allele. P175 cells were exposed to BrdU for 14 or 5 hours. Cultures were harvested for mitotic cells and processed for ReTiSH. A–H) Mitotic spreads were hybridized simultaneously with either two or three different FISH probes. First, each ReTiSH assay included a chromosome 6 centromeric probe, which hybridizes to both chromosome 6 s at both time points. Arrows mark the centromeric signals in each panel. P175 cells contain a chromosome 6 centromeric polymorphism so that one of the chromosomes displays a much larger FISH signal (*). Each assay included BAC probes representing asynchronously replicating loci on chromosome 6. The insets in each panel show the two chromosome 6 s aligned at their centromeres. A and B) ReTiSH assay using an *ASAR6* BAC (RP11-374I15; red) plus a chromosome 6 centromeric probe (green). The *ASAR6* BAC hybridized to the chromosome 6 with the larger centromere at the 5 hour time point. C and D) ReTiSH assay using an *ASAR6* BAC (RP11-374I15; green) plus a probe to the Aprt transgene (red), which is integrated into only one of the chromosome 6 s [9]. The Aprt transgene is present on the chromosome 6 with the larger centromeric signal (and see Table 3). E and F) ReTiSH assay using a *FUT9* BAC (RP11-75N19; green) plus a chromosome 6 centromeric probe (red). The *FUT9* BAC hybridized to the chromosome 6 with the larger centromere at the 5 hour time point. G and H) ReTiSH assay using an *ASAR6* BAC (RP11-374I15; green) plus a *ME1* BAC (RP11-703G14; red). *ASAR6* and *ME1* show hybridization signals on the opposite chromosome 6 s at the 5 hour time point.

All mono-allelically expressed genes share the property of asynchronous replication. Typically, the earlier-replicating alleles of these mono-allelically expressed genes are the expressed alleles. However, one unusual characteristic of the *XIST* gene is that the silent allele on the active X chromosome replicates before the expressed allele on the inactive X chromosome [14]. Therefore, to determine if *ASAR6* shares this property with *XIST* we analyzed the asynchronous replication of *ASAR6* in cells where the active and inactive alleles could be identified. For this analysis we used the ReTiSH assay in the clonal cell line P175, where *ASAR6* expression is mono-allelic and the actively transcribed chromosome for *ASAR6* contains a transgene insertion [9]. To confirm that the ReTiSH assay can detect asynchronous replication in P175 cells we first analyzed the hybridization patterns of the rDNA loci. As expected, we detected hybridization of an rDNA probe to all of the rDNA containing chromosomes at the 14 hour time point, but hybridization of the rDNA probe to only 5 chromosomes, representing single copies of chromosomes 13, 14, 15, 21, and 22, at the 5 hour time point (Figure S3A and S3B). In addition, P175 cells contain a centromeric polymorphism on chromosome 15, which allowed for an unambiguous distinction between the two homologs. Thus, using a centromeric probe to chromosome 15 we found that the rDNA probe hybridized to the smaller centromere containing chromosome 15 at the 5 hour time point, indicating that the chromosome with the smaller centromere contains the later replicating rDNA allele (Figure S3C and S3D). Therefore, the ReTiSH assay allows us to identify the earlier and later replicating alleles of loci on homologous chromosomes in P175 cells. Next, to identify the earlier and later replicating alleles of *ASAR6* we used a similar two-color FISH assay to detect replication of *ASAR6* plus other loci along chromosome 6. For this analysis we also took advantage of a chromosome 6 centromeric polymorphism, which allowed for an unambiguous distinction between the two chromosome 6 homologs in P175 cells. Table 3 shows the results of this analysis and indicates that the later replicating allele of *ASAR6* (FISH positive at the 5 hour time point) is linked to the chromosome 6 with the larger centromere (Figure 3A and 3B; and Table 3). In addition, the larger chromosome 6 centromere is linked to the Aprt transgene (Figure 3C and 3D; and Table 3). Because the Aprt transgene is inserted in the chromosome 6 that expresses *ASAR6* [9], we conclude that *ASAR6* is expressed from the later replicating allele. A similar analysis of the asynchronous replication timing of the protein-coding gene *FUT9* indicated that the later replicating allele is also linked to the larger centromere of chromosome 6 (Figure 3E and 3F; and Table 3). These data indicate that the asynchronous replication of the closely linked genes *ASAR6* and *FUT9* is coordinated in *cis* in P175 cells. However, because *FUT9* is expressed from the opposite chromosome as *ASAR6* [9], *FUT9* is expressed from the earlier replicating allele. Furthermore, ReTiSH assays on additional asynchronous loci indicated that asynchronous replication along chromosome 6 is coordinated both in *cis* and in *trans* in P175 cells (Figure 3E–3H; Figure S3E and S3F; and Table 3), and these data are consistent with our observations in primary skin fibroblasts (Figure 1H, Table 2; and [9]) and PBLs (Figure S2; and Table 3).

### *ASAR6* is mono-allelically expressed in primary blood lymphocytes

**Figure 4:**
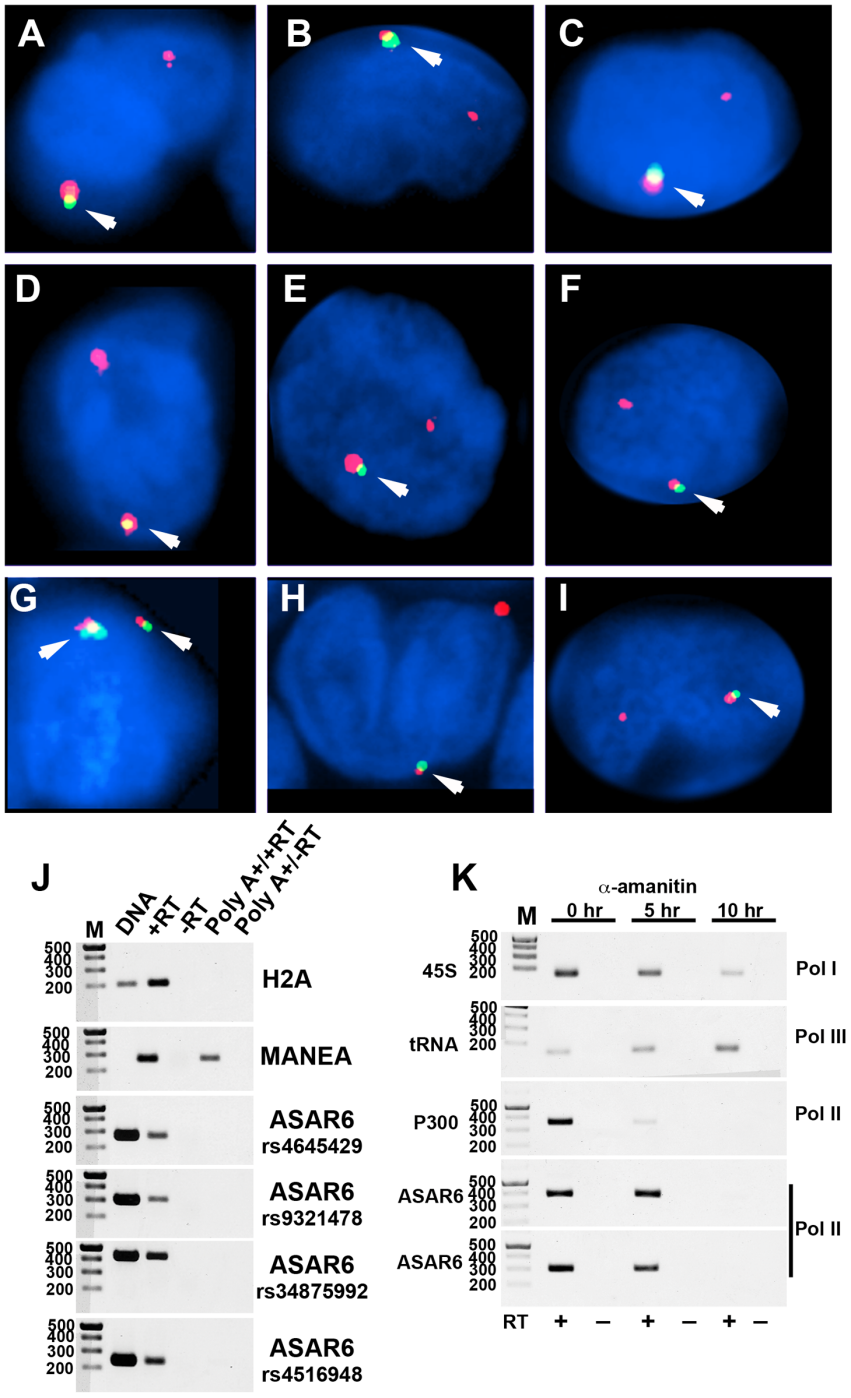
Mono-allelic expression of *ASAR6* in human PBLs. A–I). RNA-DNA FISH for expression of *ASAR6*. PBLs were subjected to RNA FISH (green) using a Fosmid (G248P86031A6) probe for *ASAR6*. Slides were subsequently re-fixed and processed for DNA FISH (red) using BAC RP11-959I6, located distal to *FUT9* (BAC#4 in Figure 1H). In regions of the slide where the FISH worked well, the RNA FISH probe detected a positive signal in >80% of the cells. DNA was stained with DAPI. Arrows mark the location of the RNA signals. Two sites of RNA hybridization were detected in <5% of cells (see Figure S4 for each probe in separate images). Panel G shows an example of bi-allelic expression of ASAR6. J) *ASAR6* RNA is not polyadenlylated. Total RNA extracted from P175 cells was subjected to two rounds of Poly A selection followed by reverse transcriptase reactions (RT) in the presence (+) or absence (−) of reverse transcriptase followed by semi-quantitative PCR using primers to histone H2A, *MANEA*, or *ASAR6*. Genomic DNA was used as positive control. The primers used to detect *MANEA* cDNA span an intron and therefore do not result in a product in the genomic DNA lane. Four independent PCRs, from four different regions of ASAR6 (each spanning a different SNP; rs4645429, rs9321478, rs34875992, rs4516948) were used to detect ASAR6 RNA. The sizes of the DNA ladder (M) are indicated in base pairs. K) *ASAR6* is transcribed by RNA Polymerase II. P175 cells were exposed to 20 ug/mL of α-amanitin for 0, 5, and 10 hours. Total RNA was subjected to reverse transcriptase reactions (RT) in the presence (+) or absence (−) of reverse transcriptase followed by semi-quantitative PCR using primers to 45S RNA, a tRNA, *P300* and *ASAR6*. The sizes of the DNA ladder (M) are indicated in base pairs. Two independent PCRs, from two different regions of ASAR6 (associated with SNPs rs34875992 [top] and rs9321478 [bottom]) were used to detect ASAR6 RNA.

We previously detected mono-allelic expression of *ASAR6* in immortalized human lymphoblastoid cell lines, which involved the isolation of proliferating clones derived from single cells [9]. These clonal cell lines expressed either the maternal or the paternal allele of *ASAR6*, indicating that *ASAR6* is subject to random mono-allelic expression. In order to determine if *ASAR6* is mono-allelically expressed in primary human tissues, we used RNA-DNA FISH to assay expression of *ASAR6* in primary cultures of human PBLs. We detected single sites of RNA hybridization in >95% of cells expressing *ASAR6* (Figure 4A–4I; and see Figure S4 for each probe in separate images). In addition, the hybridization signal detected from *ASAR6* is limited to a relatively small region of the nucleus, indicating that ASAR6 RNA does not coat chromosome 6 in PBLs.

During our original characterization of ASAR6 RNA, using RT-PCR assays with primers that spanned many different regions of *ASAR6* covering ∼200 kb of genomic DNA, we failed to detect any evidence for the presence of introns ([9]; and data not shown). In addition, to determine if ASAR6 RNA is polyadenylated we subjected total RNA to two rounds of Poly A selection and subsequently assayed ASAR6 RNA by RT-PCR. Figure 4J shows that ASAR6 RNA was not detected in the Poly A+ fraction. Similarly, RNA expressed from the non-polyadenylated RNA for histone H2A was also not detected in the Poly A+ fraction. In contrast, RNA from the protein-coding gene *MANEA* was detected in the Poly A+ fraction. Therefore, ASAR6 RNA is not polyadenylated. Furthermore, cell fractionation studies indicated that ASAR6 RNA is restricted to the nuclear compartment (E.P.S. and M.J.T. data not shown), which is consistent with our RNA-DNA FISH analysis (Figure 4A–4I; and [9]). Consistent with these observations, RNA-seq data from the Encode project found that expression of ASAR6 RNA is enriched in the nuclear Poly A- fraction [[25]; and see Figure S5B and S5C].

The observations described above suggest that ASAR6 RNA does not contain introns nor does it contain a Poly A tail, which raises the question of which RNA polymerase is responsible for transcribing *ASAR6*. Therefore, to determine if ASAR6 RNA is the product of RNA Polymerase II we treated cells with α-amanitin, which is a selective inhibitor of RNA Polymerase II [26], and assayed expression of ASAR6 RNA using a semi-quantitative RT-PCR assay. The results of this analysis are shown in Figure 4K, and indicate that ASAR6 RNA is indeed sensitive to α-amanitin. Similarly, RNA expressed from the protein-coding gene *P300* is also sensitive to α-amanitin treatment. In contrast, expression of 45S RNA (an RNA Polymerase I product) and a tRNA (an RNA Polymerase III product) was not inhibited by α-amanitin. We conclude that *ASAR6* is transcribed by RNA Polymerase II. In addition, this analysis indicated that the half-life of ASAR6 RNA is approximately 5 hours, and is much longer than the half-life of P300 RNA (∼2 hours), which is a spliced and polyadenylated RNA Polymerase II product. Thus, ASAR6 RNA represents an unusual RNA Polymerase II product that it is not spliced, not polyadenlyated, yet is not rapidly degraded [27].

### Delineation of the critical sequences of *ASAR6* required for replication delay of chromosome 6

We previously found that nested deletions in chromosome 6, originating from an integrated loxP cassette in the P175 cell line, resulted in delayed replication timing of chromosome 6 [9]. This analysis indicated that deletions ranging in size from ∼30 mb to as small as ∼76 kb resulted in delayed replication. Interestingly, all of the deletions that cause delayed replication result in the removal of the 5′ region of the *ASAR6* gene (see Figure S5A). Therefore, we next determined if a smaller deletion, which would leave the 5′ end of *ASAR6* intact, would result in DRT. For this analysis we used homologous recombination, using a rAAV gene targeting strategy [28], to introduce a second loxP site into chromosome 6 of P175 cells. Subsequent Cre expression resulted in the deletion of ∼47 kb upstream of *ASAR6* (see Figure S6).

**Figure 5:**
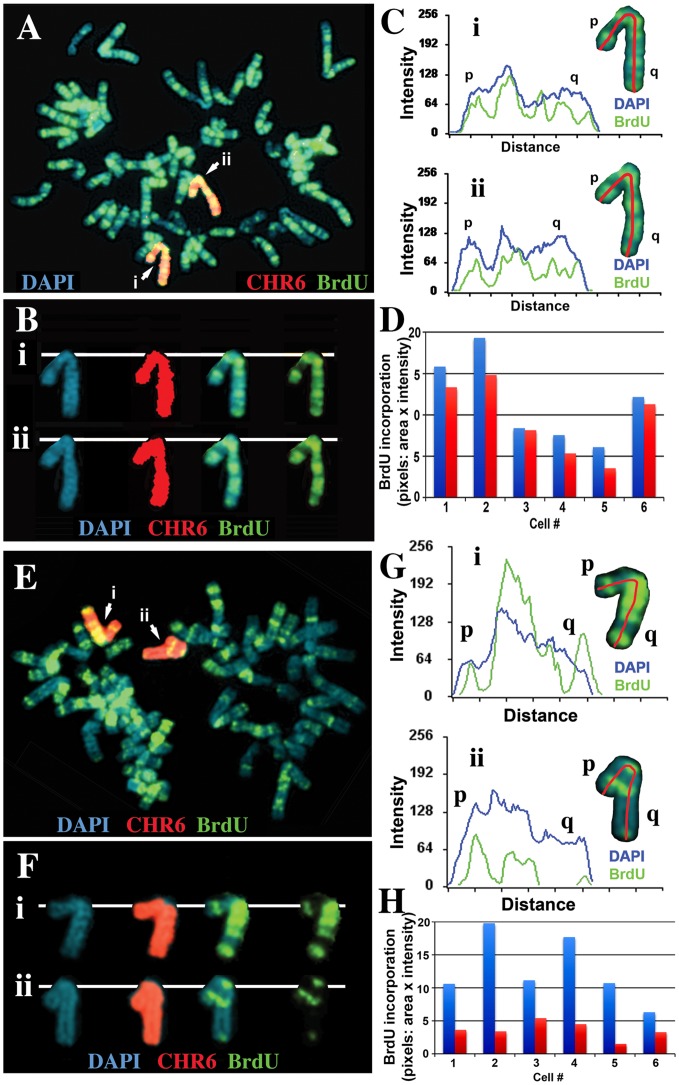
Delineation of the critical region required for DRT on chromosome 6. Cells containing either an ∼47 kb deletion (ΔAAV-1d) or an ∼76 kb deletion (Δ175-23a) at the *ASAR6* locus (see Figure 7A and S5A) were incubated with BrdU for 4.5 hours, harvested, and processed for FISH using a chromosome 6 paint (red) as probe and for BrdU incorporation using an antibody against BrdU (green). The DNA was stained with DAPI (blue). A) An example of this analysis from the ΔAAV-1d (∼47 kb deletion). The two chromosome 6 s are indicated with arrows, and arbitrarily assigned i or ii. B) The two chromosome 6 s from panel A, were cut out and aligned with each color displayed separately or in combination. C) Pixel intensity profiles of the BrdU incorporation (green), and DAPI (blue) staining along the two chromosome 6 s from panel A. D) Quantification of the BrdU incorporation in chromosome 6 s. The red and blue bars represent the two chromosomes identified by the chromosome 6 paint in six different cells. E) An example of this analysis from the Δ175-23a cells (∼76 kb deletion). The two chromosome 6 s are indicated with arrows, and arbitrarily assigned i or ii. F) The two chromosome 6 s from panel E, were cut out and aligned with each color displayed separately. G) Pixel intensity profiles of the BrdU incorporation (green), and DAPI (blue) staining along the two chromosome 6 s from panel E. H) Quantification of the BrdU incorporation in chromosome 6 s. The red and blue bars represent the two chromosomes identified by the chromosome 6 paint in six different cells, the late replicating chromosome contains a deletion of ∼76 kb [9].

We next assayed the replication timing of the chromosome 6 s present in two independent isolates of cells containing this ∼47 kb deletion in chromosome 6. Cultures were incubated with BrdU for 4.5 hours and mitotic cells were harvested, processed for BrdU incorporation and subjected to FISH using a chromosome 6 paint as probe. Comparing the BrdU incorporation patterns between chromosome 6 s in multiple cells indicated that the two copies of chromosome 6 incorporated similar amounts of BrdU, indicating that the chromosome 6 s containing this relatively small deletion retain normal replication timing (Figure 5A–5D). In contrast, a similar analysis of cells containing a larger ∼76 kb deletion showed a large difference in BrdU incorporation, consistent with a delay in replication timing of >2 hours of one of the chromosome 6 s (Figure 5E–5H). Therefore, these two deletions define the critical region required for the DRT phenotype of chromosome 6, and represents ∼29 kb of genomic DNA containing the 5′ region of *ASAR6* (see Figure S5A).

### Disruption of *ASAR6* results in instability of chromosome 6

**Figure 6:**
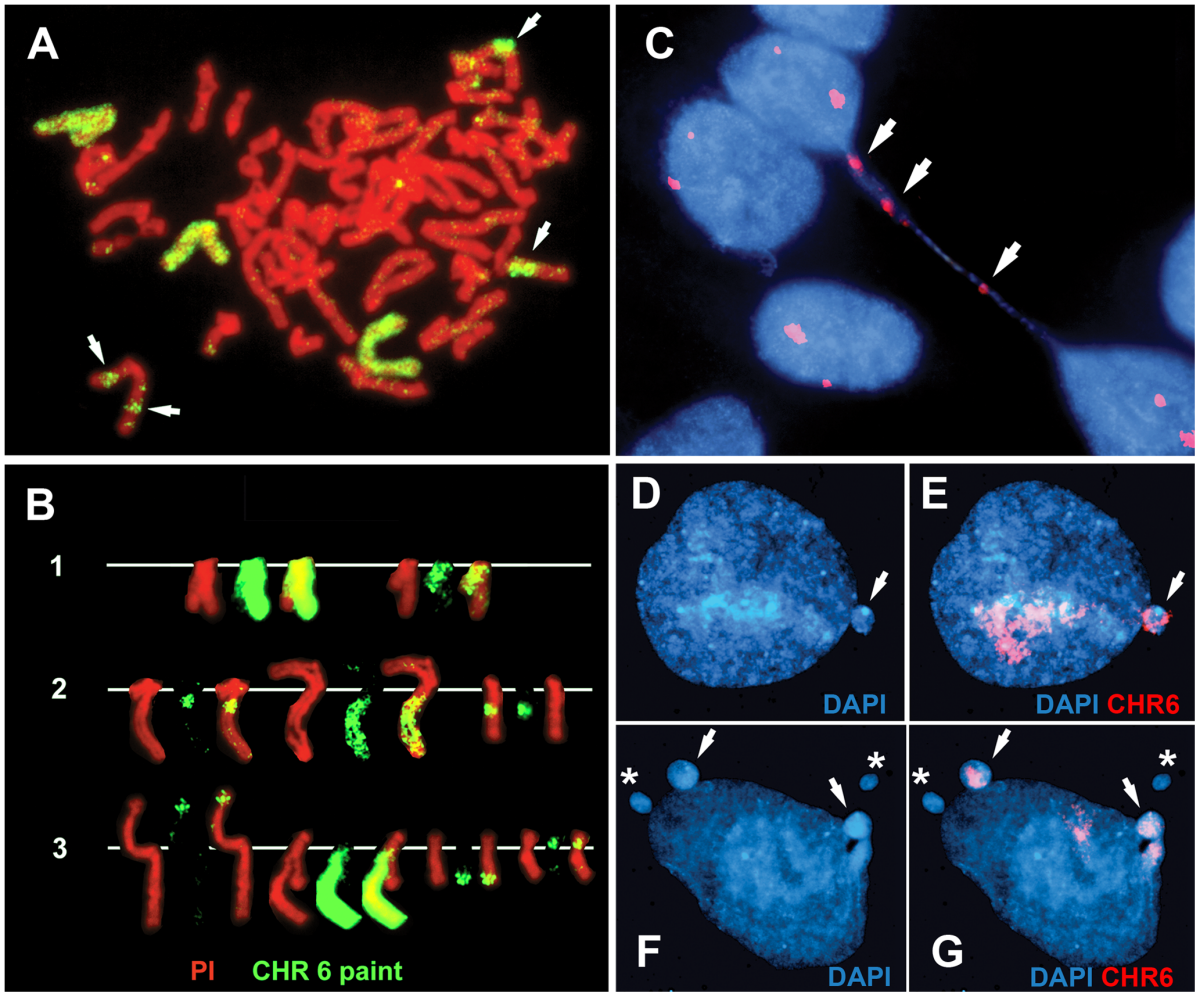
Disruption of *ASAR6* leads to instability of chromosome 6. (A) An example of a mitotic spread containing three different translocations involving chromosome 6. Cells containing an engineered deletion of *ASAR6* were processed for FISH using a chromosome 6 paint as probe (green). The DNA was stained with propidium iodide. The arrows mark the locations of three different rearrangements involving chromosome 6. B) Partial karyotypes from three different mitotic spreads (1–3). The DNA was stained with propidium iodide. Notice that each cell contained different rearrangements involving chromosome 6. C) An example of a bridged chromosome between two daughter nuclei. Cells containing a disruption of ASAR6 were processed for FISH using a chromosome 6 centromeric probe (red). The DNA was stained with DAPI. Arrows mark the sites of hybridization to the chromosome 6 centromeric probe on the bridged chromosome. Chromosome bridges involving chromosome 6 were detected in approximately 1/500 metaphase spreads. D–G) Examples of micronuclei derived from chromosome 6. Cells containing a deletion of ASAR6 were processed for FISH using a chromosome 6 paint as probe (red). The DNA was stained with DAPI. Arrows mark micronuclei that hybridized to the chromosome 6 probe. Asterisks mark micronuclei that did not hybridize to the chromosome 6 paint.

We previously found that chromosomes with DRT/DMC cause a 30–80 fold increase in the rate at which secondary rearrangements occur on the affected chromosome, indicating that DRT/DMC causes genomic instability [8]. During the characterization of chromosomes containing deletions of *ASAR6* we noticed numerous secondary rearrangements affecting chromosome 6. Figure 6A and 6B show examples of these secondary rearrangements in cells with an engineered deletion of *ASAR6*. In addition, chromosome 6 was occasionally observed as a bridged chromosome between two daughter nuclei (Figure 6C). Secondary rearrangements and bridged chromosomes involving chromosome 6 were not detected in cultures of P175 cells or in subclones of P175 cells in the absence of *ASAR6* disruption (not shown; and see [8]). In addition, we found that cells containing a deletion of the *ASAR6* gene show a 10 fold increase in the frequency of micronuclei that hybridize to a chromosome 6 paint probe [0.5% (5/1000) of cells prior to deletion and 5.0% (50/1000) after deletion] (Figure 6D–6G). Furthermore, we also detected micronuclei that did not hybridize with the chromosome 6 paint in cells that also contained chromosome 6 positive micronuclei (Figure 6F and 6G). Because chromosome 6 DNA is often translocated to other chromosomes in these *ASAR6* deleted clones (Figure 6A and 6B), and these secondary rearrangements can also display DRT/DMC [8], we could not determine if the micronuclei that do not hybridize with the chromosome 6 paint (Figure 6G) did not originate from one of these secondary rearrangements with DRT/DMC. Regardless, these data indicate that DNA from other chromosomes within the same cells as a DRT/DMC chromosome can also be segregated into micronuclei. These observations indicate that *ASAR6* functions to maintain structural integrity and proper mitotic segregation of chromosome 6 via a *cis* acting mechanism, and that the instability induced by DRT/DMC on one chromosome can affect the stability of other chromosomes within the same cell.

### Ectopic integration of an *ASAR6* transgene delays replication timing of mouse chromosomes

**Figure 7:**
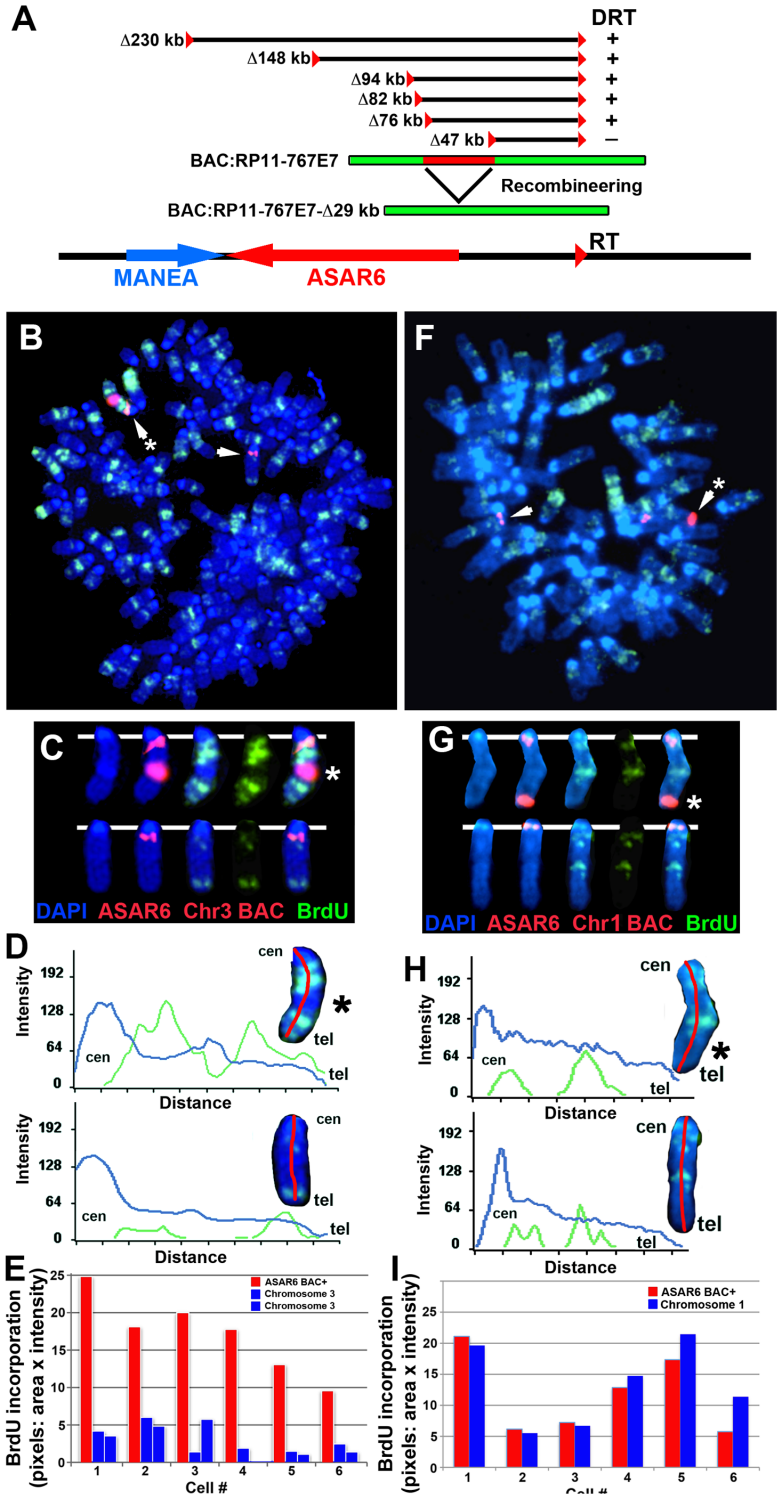
Ectopic integration of *ASAR6* results in delayed replication of mouse chromosomes. A) Schematic representation of the *ASAR6* BAC (RP11-767E7) used for integration into mouse chromosomes. The approximate locations of the loxP integration sites (red triangles) in P175 cells, deletions [in kilobases (Δkb)], and the location of the ASAR6 BAC (green) are indicated. The ASAR6 BAC was modified using recombineering to contain a deletion of the ∼29 kb (red) critical region identified by our deletion analysis. B–E) Cells containing a multicopy array of the *ASAR6* BAC integrated into mouse chromosome 3 were incubated with BrdU for 2.5 hours, harvested for mitotic cells, and processed for FISH using the *ASAR6* BAC (RP11-767E7) plus a mouse chromosome 3 BAC (RP23-430A13) as probes (both in red), and for BrdU incorporation using an antibody against BrdU (green). The DNA was stained with DAPI (blue). The chromosome 3 s are indicated by arrows. C) The chromosome 3 s from panel B, were cut out and aligned with each color displayed separately or in combination. D) Pixel intensity profiles of the BrdU incorporation (green), and DAPI (blue) staining along the chromosome 3 s from panel B are shown. Note that the chromosome 3 with the ASAR6 BAC integration (*) contains much more BrdU incorporation, indicating that it displays delayed replication. E) Quantification of the BrdU incorporation in six different cells. The red bars represent the chromosome containing the ASAR6 BAC and the blue bars represent the normal chromosome 3 s in six different cells. Most of the cells in this clone contained three chromosome 3 s. The values represent the total number of pixels (area×intensity)×1000. F–I) Cells containing a multicopy array of the *ASAR6* BAC containing an ∼29 kb deletion (see panel A, RP11-767E7-Δ29 kb) integrated into mouse chromosome 1 were incubated with BrdU for 3.0 hours, harvested for mitotic cells, and processed for FISH using the *ASAR6* BAC (RP11-767E7) plus a mouse chromosome 1 BAC (RP23-34K7) as probes (both in red), and for BrdU incorporation using an antibody against BrdU (green). The DNA was stained with DAPI (blue). The chromosome 1 s are indicated with arrows. The chromosome with the *ASAR6* BAC is marked with an asterisk (*). G) The chromosome 1 s from panel F, were cut out and aligned with each color displayed separately or in combination. H) Pixel intensity profiles of the BrdU incorporation (green), and DAPI (blue) staining along the chromosome 1 s from panel F are shown. Note that the chromosome 1 containing the ASAR6 BAC (Δ29 kb) shows similar BrdU incorporation as the normal chromosome 1. I) Quantification of the BrdU incorporation in six different cells. The red bars represent the chromosome containing the ASAR6 BAC (Δ29 kb) and the blue bars represent the normal chromosome 1 s in six different cells. The values represent the total number of pixels (area×intensity)×1000.

One well-characterized activity of the *Xist* gene is its ability to delay DNA replication timing in *cis* when ectopically integrated into chromosomes [reviewed in [29]. This activity is not restricted to ES cells, as ectopic integration of either human or mouse *XIST/Xist* into the chromosomes of differentiated mammalian cell lines can delay replication of entire chromosomes [30], [31], [32], [33]. Therefore, to determine if *ASAR6* also displays this activity we tested whether ectopic integration of cloned genomic DNA from the *ASAR6* locus can cause delayed replication timing of mouse chromosomes. For this analysis we used a BAC that contains ∼180 kb of genomic DNA spanning the critical region of the *ASAR6* locus required for DRT (see Figure 7A and Figure S5A). Prior to transfection, the BAC was modified by recombineering [34] to contain a Hygromycin B resistance gene to enable positive selection in mammalian cells. Mouse cells were transfected with the modified BAC and subjected to selection in media containing Hygromycin B. Individual clones were isolated and analyzed for BAC integration sites, BAC copy number, and replication timing of the affected chromosome.

Figure 7B shows the replication timing analysis of a clone containing a multicopy array (∼20 copies) of the *ASAR6* BAC integrated into mouse chromosome 3. Cultures were incubated with BrdU for 2.5 hours and mitotic cells were harvested, processed for BrdU incorporation and subjected to FISH using a mouse chromosome 3 BAC (located near the centromere) plus the *ASAR6* BAC as probes. The FISH signal from the chromosome 3 centromeric region allowed us to identify all of the chromosome 3 s, and the presence or absence of the *ASAR6* BAC allowed us to distinguish between the integrated and non-integrated chromosomes, respectively. Comparing the BrdU incorporation pattern between chromosome 3 s in multiple cells indicated that the chromosome containing the *ASAR6* BAC was delayed in replication timing (Figure 7B–7E). Delayed replication was also detected in a second clone containing ectopic integration of the *ASAR6* BAC into a different mouse chromosome (data not shown), indicating that integration of the *ASAR6* BAC into different mouse chromosomes results in delayed replication.

Our chromosome engineering studies created a set of nested deletions at the *ASAR6* locus and defined an ∼29 kb region of *ASAR6* DNA, that when deleted, results in DRT/DMC on human chromosome 6 (see Figure 7A and Figure S5A). To determine if this critical region is also required for delayed replication following ectopic integration, we deleted this ∼29 kb region from the *ASAR6* BAC (see Figure 7A) using recombineering strategies and reintroduced the deleted BAC into mouse chromosomes. Figure 7F–7I shows the results of the replication timing analysis on a clone containing ∼20 copies of the deleted BAC integrated into mouse chromosome 1. This analysis indicated that the integrated chromosome 1 does not display delayed replication. An analysis of similar integrations of the deleted BAC into three other mouse chromosomes similarly did not display delayed replication (data not shown). In total, we detected delayed replication in 2 out of 3 ectopic integrations of the intact *ASAR6* BAC and in 0 out of 4 ectopic integrations of the deleted BAC. While it is not possible to conclude that the BAC with the ∼29 kb deletion cannot induce delayed replication upon ectopic integration, especially with the limited number of integrations assayed, our observations suggest that the ∼29 kb critical region of *ASAR6* is necessary to prevent DRT in its native location on chromosome 6 and is sufficient to cause DRT when integrated at ectopic locations.

## Discussion

Mammalian cells replicate their genomes every cell cycle during a defined replication-timing program. It is clear that the determinants of replication timing are not encoded within the sequence of the origins of replication, but rather the timing of origin firing is dictated by chromosomal location [35], [36]. Recent studies indicate that at least half of the genome is subject to changes in the temporal sequence of DNA replication during development [37], [38]. The current thinking is that replication timing is directly linked to complex higher-order features of chromosome architecture [39], [40]. However, both the mechanisms and the significance of this temporal replication program remain poorly defined.

Asynchronous replication represents an epigenetic state that is established early in development, and is not dependent on gene expression [17], [19], [20]. An additional feature of many autosomal mono-allelic genes is that they do not appear separately in the genome, but rather they appear in groups that occupy relatively large chromosomal domains [18]. We found that *ASAR6* resides within an ∼1.2 mb domain of *cis*-coordinated asynchronous replication timing, which includes at least five other genes located at 6q16.1. In addition, we found that *ASAR6* is expressed from the later replicating allele, which is in contrast to other mono-allelically expressed genes located within this same domain.

Previous studies have shown that most if not all of the genes residing on the inactive X chromosome display late replication [14], [41], [42], [43], indicating that the asynchronous replication of X linked genes in female cells is coordinated in *cis*. The observations described here are consistent with previous reports showing that, like X chromosomes, autosome pairs also display coordination in their asynchronous replication of random mono-allelically expressed genes [17], [20], [23]. In this report we found that human chromosome 6 displays coordinated asynchronous replication between loci separated by >87 mb of genomic DNA, and between loci on either side of the centromere. However, we found that chromosome 6 contains loci that display both *cis* and *trans* coordination of asynchronous replication. Another important feature of asynchronous replication is that it can be observed in all cell types. Thus, we found that chromosome 6 displays both *cis* and *trans* coordination of asynchronously replicating loci in two different human cell types, primary skin fibroblasts and PBLs [also see [9]]. These observations represent the first example of a mammalian chromosome displaying both *cis* and *trans* coordination of asynchronous replication.

In general, early replicating regions of the genome are correlated with transcriptional activity. While parallels between X inactivation and the *cis* coordinated replication asynchrony of autosomal mono-allelic genes have been made [17], [18], [20], [23], [44], [45], previous reports found that not all random mono-allelically expressed genes are expressed from the same homolog [15], [16], [46]. One obvious presumption from these studies is that the later replicating alleles of some of these mono-allelic genes represent the actively transcribed alleles. However, the asynchronous replication of these ‘non-coordinated’ mono-allelically expressed genes was not assayed in these earlier studies. Therefore, our results suggest a possible explanation for the apparent ‘non-coordinated’ expression pattern of autosomal mono-allelic genes. Thus, it is possible that all autosomes display a pattern of *cis* and *trans* coordination in asynchronous replication similar to human chromosome 6. Therefore, the apparent non-coordinated expression pattern of some autosomal mono-allelic genes may still be correlated with earlier and later replicating alleles. Therefore, a detailed analysis of the coordinated asynchronous replication in combination with allele-specific expression assays within the same cells is required to determine if mono-allelic expression is indeed originating from the later replicating alleles.

The observation of *trans* coordination of asynchronous replication on chromosome 6 indicates that there is a reciprocal relationship between the asynchronous replication of certain chromosome 6 loci so that their earlier replicating alleles are always on the same homolog as the later replicating alleles for other loci. This reciprocal relationship appears to affect the entire chromosome, as *trans* coordination is observed with loci that are on either side of the chromosome 6 centromere. The reason for this reciprocal relationship between these asynchronously replicating loci is currently not known. One possibility is that asynchronous replication of certain genes may be the consequence of *cis* acting chromosomal elements that reside within the same chromosome domain. These *cis* acting elements may have functions, e.g. regulating chromosome-wide replication timing, that don't involve transcriptional regulation directly but do require asynchronous replication for their activity. Thus, a gene located within the same domain as one of these *cis* acting elements may simply be responding to the late replication of the domain, and consequently transcriptional silencing on one allele may be a secondary affect of the epigenetic state associated with the later replicating domain.

Previous work indicates that the *ASAR6* and *Xist* genes share many characteristics, including: 1) they both express large non-coding RNAs, 2) they both display random mono-allelic expression, 3) they both display asynchronous replication that is coordinated with other linked mono-allelic genes, 4) disruption of either gene results in delayed replication timing and instability of entire chromosomes in *cis*, and 5) disruption of either gene results in the transcriptional activation of the previously silent alleles of linked mono-allelic genes [9], [10], [12], [13]. In addition, in this report we found that *ASAR6* is expressed from the later replicating allele, which is also a feature of *Xist* [14]. Furthermore, another well-characterized activity contained within the *Xist* gene is the ability to delay replication timing of entire chromosomes upon ectopic integration of cloned genomic DNA [reviewed in [29]]. In this report we found that ectopic integration of cloned genomic DNA containing *ASAR6* also has the ability to delay replication of mouse chromosomes following ectopic integration. Furthermore, we found that the ability of *ASAR6* transgenes to delay replication at ectopic locations occurred only when multiple copies of the transgene were integrated, which was also observed with *Xist* genomic transgenes [30], [33], [47], [48], [49]. However, there are also some notable differences between A*SAR6* and *Xist*. For example, the *Xist* non-coding RNA physically “coats” the inactive X chromosome, while the *ASAR6* RNA does not appear to coat chromosome 6 in adult somatic tissues [9]. In addition, *Xist* RNA is expressed in all female adult tissues (reviewed in [10], [11]), while expression of *ASAR6* is limited to only a subset of adult tissues [9]. Furthermore, we found that even though *ASAR6* RNA is transcribed by RNA Polymerase II it is not polyadenlyated and does not appear to contain introns, which is in contrast to the polyadenlyated and spliced *Xist* RNA [50]. Additional studies suggest that Xist RNA mediates silencing and late replication of the inactive X chromosome via direct interactions with chromatin associated RNA binding proteins [51], [52], [53]. Additional studies designed to interrogate whether or not ASAR6 RNA interacts with these RNA interacting proteins should reveal whether or not *ASAR6* also shares these activities.

We previously found that chromosomes with DRT/DMC cause a 30–80 fold increase in the rate at which secondary rearrangements occur on the affected chromosome, indicating that DRT/DMC causes genomic instability [5], [8]. In addition, we found that chromosomes with DRT/DMC are delayed in their recruitment of Aurora B kinase, are frequently unattached to the mitotic spindle, activate the spindle assembly checkpoint, and are often seen as lagging chromosomes during mitosis [6]. In this report we found that disruption of *ASAR6* leads to several abnormalities involving chromosome 6, including: chromosomal bridges between daughter nuclei, segregation into micronuclei, and numerous secondary rearrangements. Thus, we propose a model for the instability of individual chromosomes that includes: 1) delayed replication timing of individual chromosomes caused by genetic disruption of *cis*-acting loci (e.g. *ASAR6* or *Xist*); 2) delayed recruitment of Aurora B kinase resulting in delayed mitotic chromosome condensation; 3) delayed mitotic spindle attachment leading to chromosome missegregation, bridged chromosomes, and the formation of micronuclei; 4) the onset of mitotic chromosome condensation prior to the completion of DNA synthesis leading to stalled replication forks; and 5) multiple rearrangements generated at the stalled replication forks via replicative mechanisms (Figure S7; also see [1]).

Recently, the phenomenon of “chromothripsis” was described as a new mechanism for generating complex chromosome rearrangements in cancer cells [54]. Chromothripsis appears to be a cataclysmic event in which one or a few chromosomes are fragmented and then reassembled in a haphazard manner. The authors of the original chromothripsis paper proposed that either exposure to IR or telomere dysfunction was responsible for the multiple rearrangements affecting individual chromosomes [54]. Alternatively, recent work from Dr. David Pellman's lab suggests that missegregation of chromosomes, caused by transient mitotic spindle disruption, can result in lagging chromosomes, micronucleus formation, late replication, and ‘pulverization’ of individual chromosomes [55]. In addition, the complex chromosome rearrangements associated with “genomic disorders” in humans were recently found to resemble chromothripsis [56], [57]. Sequencing the breakpoints at the complex rearrangements identified characteristic features, including small templated insertions of nearby sequences and microhomologies, suggestive of replicative processes [56]. Thus, sequencing the genomes of cells containing disruption of *ASAR6* should reveal whether or not the mechanisms that are functioning to generate rearrangements on DRT/DMC chromosomes are similar or distinct from those responsible for chromothripsis-type rearrangements.

The DRT/DMC phenotype has been detected on chromosome rearrangements involving many different human and mouse chromosomes [5], [7], [8], [9], [13]. Therefore, it seems likely that all mammalian chromosomes contain loci that function to regulate chromosome-wide replication timing of individual chromosomes. Given the similarities in structure and function of the two loci characterized to date, *Xist* and *ASAR6*, we propose that all mammalian chromosomes contain ‘inactivation/stability centers’ that function to maintain proper replication timing, mitotic chromosome condensation, mono-allelic gene expression and stability of individual chromosomes. Under this scenario every mammalian chromosome contains four distinct types of *cis*-acting elements, origins of replication, centromeres, telomeres, and ‘inactivation/stability centers”, all functioning to ensure proper replication, segregation and stability of individual chromosomes.

## Materials and Methods

### Cell culture

Low passage primary human skin fibroblasts were obtained from ATCC and cultured in DMEM plus 10% fetal bovine serum (Hyclone). Primary blood lymphocytes were isolated after venipuncture into a Vacutainer CPT (Becton Dickinson, Franklin Lakes, NJ) per the manufacturer's recommendations and grown in 5 mL RPMI 1640 (Life Technologies) supplemented with 10% fetal bovine serum (Hyclone) and 1% phytohemagglutinin (Life Technologies). P175 cells are a human *APRT* deficient cell line derived from the HT1080 fibrosarcoma [58], and were grown in DMEM (Gibco) supplemented with 10% fetal bovine serum (Hyclone). P175 derivatives were grown as above with the addition of 500 mg/ml Geneticin (Gibco), 200 mg/ml Hygromycin B (Calbiochem), and/or 10 ug/ml Blasticidin S HCl (Invitrogen). The deletion-line derivatives were grown in DMEM supplemented with 10% dialyzed fetal bovine serum (Hyclone), 10 mg/ml azaserine (Sigma) and 10 mg/ml adenine (Sigma) to facilitate selection for *Aprt*-expressing cells. Mouse C2C12 cells were used for the BAC integration assays, and were grown in DMEM supplemented with 10% fetal bovine serum. Clones containing BAC integrations were isolated following selection in media containing 200 mg/ml Hygromycin B. All cells were grown in a humidified incubator at 37°C in a 5% carbon dioxide atmosphere.

### DNA FISH

Trypsinized cells were centrifuged at 1,000 rpm for 10 minutes in a swinging bucket rotor. The cell pellet was re-suspended in 75 mM potassium chloride for 15–30 minutes at 37°C, re-centrifuged at 1,000 rpm for 10 minutes and fixed in 3∶1 methanol∶acetic acid. Fixed cells were added drop-wise to microscope slides to generate mitotic chromosome spreads using standard methods [59]. Slides with mitotic spreads were baked at 85°C for 20 minutes and then treated with 0.1 mg/ml RNAase for 1 hour at 37°C. After RNAase treatment, the slides were washed in 2×SSC (1×SSC is 150 mM NaCl and 15 mM sodium citrate) with 3 changes for 3 minutes each and dehydrated in 70%, 90%, and 100% ethanol for 3 minutes each. The slides were denatured in 70% formamide in 2×SSC at 70°C for 3 min and whole chromosome paints were used according to the manufacturer's recommendations and hybridization solutions (American Laboratory Technologies and Vysis). Detection of digoxigenin-dUTP probes utilized a three-step incubation of slides with sheep FITC-conjugated anti-digoxigenin antibodies (Roche) followed by rabbit FITC-conjugated anti-sheep antibodies (Roche) followed by goat FITC-conjugated anti-rabbit antibodies (Jackson Laboratories). Slides were stained with DAPI (12.5 mg/ml) or propidium iodide (0.3 mg/ml), cover slipped, and viewed under UV fluorescence with appropriate filters (Olympus).

Centromeric, BAC, and Fosmid probes: Mitotic chromosome spreads were prepared as described above. Slides were treated with RNase at 100 ug/ml for 1 h at 37°C and washed in 2×SSC and dehydrated in 70%, 90% and 100% ethanol. Chromosomal DNA was denatured at 75°C for 3 minutes in 70% formamaide/2×SSC, followed by dehydration in ice cold 70%, 90% and 100% ethanol. BAC and Fosmid DNAs were nick-translated using standard protocols to incorporate biotin-11-dUTP or digoxigenin-dUTP (Invitrogen). BAC and Fosmid DNAs were directly labeled with Cy3-dUTP, FITC-dUTP, Spectrum Orange-dUTP or Spectrum Green_dUTP (Vysis, Abbott Laboratories) using nick-translation or random priming using standard protocols. Final probe concentrations varied from 40–60 ng/*u*l. Centromeric probe cocktails (Vysis) plus BAC or Fosmid DNAs were denatured at 75°C for 10 minutes and prehybridized at 37°C for 30 minutes. Probes were applied to slides and incubated overnight at 37°C. Post-hybridization washes consisted of three 3-minute rinses in 50% formamide/2×SSC, three 3-minute rinses in 2×SSC, and finally three 3-minute rinses in PN buffer (0.1 M Na2HPO4+0.0 M NaH2PO4, ph 8.0, +2.5% Nonidet NP-40), all at 45°C. Signal detection was carried out as described [60]. Amplification of biotinylated probe signal utilized alternating incubations of slides with anti-avidin (Vector) and FITC-Extravidin (Sigma). Slides were then counterstained with either propidium iodide (2.5 ug/ml) or DAPI (15 ug/ml) and viewed under UV fluorescence (Olympus).

### RNA–DNA FISH

Cells were plated on microscope slides treated with concanavalin A (Sigma) at ∼50% confluence and incubated for 4 hours in complete media in a 37°C humidified CO_2_ incubator. Slides were rinsed 1 time with sterile RNase free PBS. Slides were incubated for 30 seconds in CSK buffer (100 mM NaCl, 300 mM Sucrose, 3 mM MgCl_2_,10 mM Pipes, ph 6.8), 5 minutes in CSK buffer plus 0.1% Triton X-100, and then for an addition 30 seconds in CSK buffer at room temperature. Cells were fixed in 4% paraformaldehyde in PBS for 10 minutes at room temperature. Slides were rinsed in 70% ETOH and stored in 70% ETOH at 4°C until use. Just prior to use, slides were dehydrated through an ETOH series (70%, 90% and 100%) and allowed to air dry. Denatured probes were prehybridized with Cot-1 DNA at 37°C for 30 min. Slides were hybridized at 37°C for 14–16 hours. Slides were washed as follows: 3 times in 50% formamide/2×SSC at 42°C for 5 minutes, 3 times in 2×SSC at 42°C for 5 minutes, 3 times in 4×SSC/0.1% Tween 20 at room temperature for 3 minutes. Slides were then fixed in 4% paraformaldehyde in PBS for 5 minutes at room temperature, and briefly rinsed in 2×SSC at room temperature. The slides were then dehydrated in 70%, 90% and 100% ETOH, and then processed for DNA FISH, including the RNAase treatment step, as described above. Slides were then counterstained with either propidium iodide (2.5 ug/ml) or DAPI (15 ug/ml) and viewed under UV fluorescence (Olympus). Z-stack images were generated using a Cytovision workstation.

### Replication timing assay

The BrdU replication timing assay was performed on exponentially dividing cultures essentially as described [61]. Briefly, asynchronously growing cells were exposed to 20 ug/ml of BrdU (Sigma) for 2.0, 2.5, 3.0 4.5, and 5 hours. Mitotic cells were harvested in the absence of colcemid, treated with 75 mM KCl for 15–30 minutes at 37°C, fixed in 3∶1 methanol∶acetic acid and dropped on wet ice cold slides. The chromosomes were denatured in 70% formamide in 2×SSC at 70°C for 3 minutes and processed for DNA FISH, as described above. The incorporated BrdU was then detected using a FITC-labeled anti-BrdU antibody (Becton Dickinson). Slides were stained with propidium iodide (0.3 mg/ml), cover slipped, and viewed under UV fluorescence. Due to the variability in the length of G2 between cells in different clones, the time point at which 50% of the mitotic cells contained BrdU incorporation was used for quantitative analysis. All images were captured with an Olympus BX Fluorescent Microscope using a 100× objective, automatic filter-wheel and Cytovision workstation. Individual chromosomes were identified with either chromosome-specific paints or centromeric probes in combination with BACs from the deleted regions. Utilizing the Cytovision workstation, each chromosome was isolated from the metaphase spread and a line drawn along the middle of the entire length of the chromosome. The Cytovision software was used to calculate the pixel area and intensity along each chromosome for each fluorochrome occupied by the DAPI and BrdU (FITC) signals. The total amount of fluorescent signal was calculated by multiplying the average pixel intensity by the area occupied by those pixels.

### ReTiSH

We used the ReTiSH assay essentially as described [23]. Briefly, unsynchronized, exponentially growing cells were treated with 30 µM BrdU (Sigma) for 6 or 5 and 14 hours. Colcemid (Sigma) was added to a final concentration of 0.1 µg/mL for 1 h at 37°C. Cells were trypsinized, centrifuged at 1,000 rpm, and resuspended in prewarmed hypotonic KCl solution (0.075 M) for 40 min at 37°C. Cells were pelleted by centrifugation and fixed with methanol-glacial acetic acid (3∶1). Fixed cells were drop gently onto wet, cold slides and allowed to air-dry. Slides were treated with 100 µg/ml RNAse A at 37°C for 10 min. Slides were rinsed briefly in d_2_H_2_0 followed by fixation in 4% formaldehyde at room temperature for 10 minutes. Slides were incubated with pepsin (1 mg/mL in 2 N HCl) for 10 min at 37°C, and then rinsed again with d_2_H_2_0 and stained with 0.5 µg/µL Hoechst 33258 (Sigma) for 15 minutes. Slides were flooded with 200 µl 2×SSC, coversliped and exposed to 365-nm UV light for 30 min using a UV Stratalinker 2400 transilluminator (Stratagene). Slides were rinsed with d_2_H_2_0 and drained. Slides were incubated with 100 µl of 3 U/µl of ExoIII (Fermentas) in ExoIII buffer for 15 min at 37°C. The slides were then processed directly for DNA FISH as described above, except with the absence of a denaturation step.

### Semi-quantitative RT–PCR

Total RNA was extracted using Trizol (Invitrogen) reagent. For Poly A selection, two rounds of selection were carried out using a PolyATract kit (Promega). Total RNA or Poly A selected RNA was subjected to reverse transcriptase reactions using Superscript III (Invitrogen) according to the manufacturers instructions. PCRs were carried out with a first cycle of 2 minutes at 95°C, 45 seconds at 58°C and 1 minute at 72°C followed by 28–30 cycles of 30 seconds at 95°C, 45 seconds at 58°C and 1 minute at 72°C. The conditions were chosen so that none of the PCRs reached a plateau at the end of the amplification protocol, i.e. they were in the exponential phase of amplification. Each set of reactions always included a genomic DNA positive control, and a no sample and a no reverse transcriptase negative controls. The PCR products were resolved on 1% agarose gels and stained with ethidium bromide. The gels were photographed under UV illumination, and the resulting image was inverted using Photoshop (Adobe).

### Recombineering

SW102 cells [62] were electroporated with 4 µg of purified BAC DNA at 1.35 kV and 600 ohms with a capacitance of 10 µF using a 0.1 cm gap cuvette. Bacterial cells were selected in 12.5 µg/mL Chloramphenicol (Cam) and 12.5 µg/mL Tetracycline (Tet) for 48 hours at 30 degrees C. To insert a Hygromycin resistance gene (Hyg^R^) into BACs we used a counter-selection modification strategy. SW102 cells are normally resistant to Streptomycin (Str). We first introduced DNA into the BAC that conferred Str sensitivity and Kanamycin (Kan) resistance to the cells and then replaced it with DNA containing Hyg^R^ and an Ampicillan (Amp) resistance gene. Upon Amp resistance the cells then revert back to Str resistance and Kan sensitivity. The PCR product used in the first recombineering step was generated by amplifying rpsL-neo template DNA (GeneBridges, Dresden, Germany) with the rpsL-neo Forward (5′-CTTATCGATGATAAGCTGTCAAACATGAGAATTGATCCGGAACCCTTAATGGCCTGGTGATGATGGCGGGATCG-3′) and rpsL-neo Reverse (5′-CCGATGCAAGTGTGTCGCTGTCGACGGTGACCCTATAGTCGAGGGACCTATCAGAAGAACTCGTCAAGAAGGCG-3′) primers. This PCR product contains 50 bp of homology to the pBACe3.6 vector on each end. Prior to electroporation, the PCR product was digested with DpnI, phenol/chloroform extracted, ethanol precipitated. Cells containing the BAC were combined with 2 µL PCR product and electroporated as mentioned above. Cells were plated on LB/agar plates containing 15 µg/mL Cam and 15 µg/mL Kan. Cam+Kan resistant cells were selected for Str sensitivity and correct targeting was confirmed by restriction enzyme digestion and PCR. To replace the rpsL-neo DNA with the Hyg^R^ gene, we used the protocol outlined above with some modifications. The PCR product used for recombination was generated by amplifying loxP-hygro-amp in pCR2.1 with Hyg Forward (5′-CCGATGCAAGTGTGTCGCTGTCGACGGTGACCCTATAGTCGAGGGACCTACAGGAAACAGCTATGACCATG-3′) and Hyg Reverse (5′-CTTATCGATGATAAGCTGTCAAACATGAGAATTGATCCGGAACCCTTAATTGTAAAACGACGGCCAGT-3′) primers. Before induction, cells were grown in 15 µg/mL Cam and 15 µg/mL Kan and following electroporation cells were plated on dishes containing 15 µg/mL Cam, 50 µg/mL Str and 15 µg/mL Amp. Correct targeting was confirmed by restriction enzyme digestion and PCR.

To make 29 kb deletion, we used a galK selection method that has been described previously (REF: PMID 15731329). Briefly, the galK cassettee was amplified with the primers galK For (5′-AAGTGTGCACATATGTGTTAGATGAAATATTGAGAAGGAACTTGAGTAAACCCTGTTGACAATTAATCATCGGCA-3′) and galK Rev (5′-TCATAATATGCATGGTAGGAAGTCTCCAGGAACTGACCCGTATAACAGGATTCAGCACTGTCCTGCTCCTT-3′). Electroporated PCR product into SW102+RP11-767E7+Hyg cells using the protocol described above. Following electroporation and recovery, cells were spun down and pellet was washed twice with M9 media and plated on M63 plates. Incubated at 32 degrees C for 6 days. Clones were grown up and analyzed for the presence of the deletion. This deletes chr6:96203250–96232818 (GRCh37/hg19).

## Supporting Information

Figure S1 ReTiSH assay on rDNA loci in primary blood lymphocytes. Human lymphocytes were obtained from normal individuals and cultured for less than one week. Cells were labeled with BrdU for 14 or 6 hours, arrested in metaphase, and subjected to ReTiSH using an rDNA probe (red). The replicated rDNA alleles were detected using a PCR fragment representing 18S rDNA, and the DNA was detected with DAPI. Arrows mark the 10 chromosomes containing rDNA clusters. A) A single metaphase spread from the 14 hour time point. B) A single metaphase spread from the 6 hour time point. C and D) The DAPI images from the 10 rDNA containing chromosomes from panels A and B were inverted and the banding patterns were used to identify the 5 rDNA containing chromosomes. C) The ReTiSH signals from a representative cell (panel A) harvested at the 14 hour time point and hybridized to the rDNA probe (red) are shown. D) The ReTiSH signals from a representative cell (panel B) harvested at the 6 hour time point and hybridized to the rDNA probe (red) are shown. The early (E) and late (L) replicating chromosomes for each pair of homologs are indicated.
(TIF)

Figure S2 Schematic diagram of chromosome 6 showing the location of the genes and loci assayed for asynchronous replication. The ∼1.2 mb region of chromosome 6 between *MANEA* and *FHL5/FHL5OST* is expanded on the right. The coordination in asynchronous replication of chromosome 6 mono-allelically expressed genes with *ASAR6* was found to be either in *cis* or in *trans*.
(TIF)

Figure S3 ReTiSH assay in P175 cells. A) Conventional DNA FISH on P175 cells using the rDNA as probe. Note that P175 cells contain 9 chromosomes that contain rDNA clusters. This is because one of the chromosome 14 s became an isochromosome [iso(14q)] and during this process a deletion of the rDNA cluster occurred. Arrows mark the chromosomes containing rDNA clusters plus the iso(14q). B) Inverted DAPI staining of the mitotic cell shown in panel A. The inverted DAPI banding pattern was used to identify chromosomes 13, 14, 15, 21, and 22. The asterisk marks the chromosome 15 containing the larger centromere polymorphism. C). 14 hour ReTiSH on P175 cells using the rDNA probe plus a chromosome 15 centromeric probe. The asterisk marks the chromosome with the larger centromeric signal. D) 5 hour ReTiSH on five representative cells probed with the rDNA (red) and chromosome 15 centromeric (green) probes. The chromosome 15 s were cut out from each cell and aligned with their centromeres on a white line. The chromosome 15 s containing the small and large centromeric polymorphism from 5 representative cells are shown. E and F) ReTiSH assay on P175 cells probed with HTRE1 (red), ME1 (green), and a chromosome 6 centromere (red). The asterisk marks the chromosome 6 with the larger centromere.
(TIF)

Figure S4 RNA-DNA FISH for expression of *ASAR6*. PBLs were subjected to RNA FISH (green) using a Fosmid (G248P86031A6) probe for *ASAR6*. Slides were subsequently re-fixed and processed for DNA FISH (red) using BAC RP11-959I6, located distal to *FUT9* (BAC#4 in Figure 1H). The four sets of panels (A–I) show the same cells used in Figure 4A–4I, except that each color is displayed separately or merged (bottom right).
(TIF)

Figure S5 Schematic illustration of the ASAR6 locus. The locations of MANEA, ASAR6, BAC RP11-767E7, the original loxP integration site [loxP(red triangle)RT] and 6 different deletions in P175 cells [9] are depicted above a screenshot of the UCSC Genome Browser of this region of chromosome 6. A) A set of nested deletions was generated in P175 cells, all except the smallest ∼47 kb deletion (Δ47) display DRT. B and C) UCSC Genome Browser view of the RNA-seq data from whole cell poly A− (B) or poly A+ (C) RNA from the human ES cell line H1 [25]. The blue tick marks indicate sequence hits from the + direction, and the red tick marks indicate sequence hits from the - direction. Note that ASAR6 RNA is enriched in the poly A− fraction, while MANEA RNA is detected in both poly A− and poly A+ fractions. The locations of 5′ caps from the Encode/RIKEN CAGE [63] track are also shown.
(TIF)

Figure S6 rAAV strategy for generating the ∼47 kb deletion upstream of ASAR6. A) Left and right arms of homology upstream of *ASAR6* were cloned into the pAAV-MCS vector (Stratagene). In addition, a loxP cassette containing the 5′ portion of the *APRT* gene (AP) plus the blasticidin resistance gene (blas^r^) are shown. B) Southern blot hybridization scheme including the location of the probe, which is outside of the homology arms used for targeting, is shown. C) Southern blot hybridization illustrating correct integration of the loxP cassette is shown. Genomic DNAs were digested with SAC1 and SPE1. Note that the loxP cassette inserts a SPE1 site into the targeted locus. Control DNAs included the parental P175, R175 [containing a t(6;10) at the original loxP site in P175 cells] and a mouse L cell somatic cell hybrid containing human chromosome 6.
(TIF)

Figure S7 Model for structural instability of individual chromosomes. Disruption of an inactivation/stability center leads to delayed replication timing of an individual chromosome. A human chromosome is depicted as a banded cylinder, and the original order of loci along the chromosome are indicated by the letters A–E. Delayed replication timing leads to delayed mitotic chromosome condensation and the onset of mitotic chromosome condensation prior to the completion of DNA synthesis (Premature condensation). This Premature condensation leads to stalled replication forks, which are depicted as X and Y structures. Multiple rearrangements (deletions, inversions, duplications, and translocations) are subsequently generated at the stalled forks via replicative mechanisms. The new order of loci are indicated with the letters E-B.
(TIF)
