# Supplementary figures and images for: Asynchronous Replication, Mono-Allelic Expression, and Long Range Cis-Effects of ASAR6
Source: PLoS Genet. 2013 Apr 4;9(4):e1003423. doi: 10.1371/journal.pgen.1003423 (PMC3617217; doi:10.1371/journal.pgen.1003423)

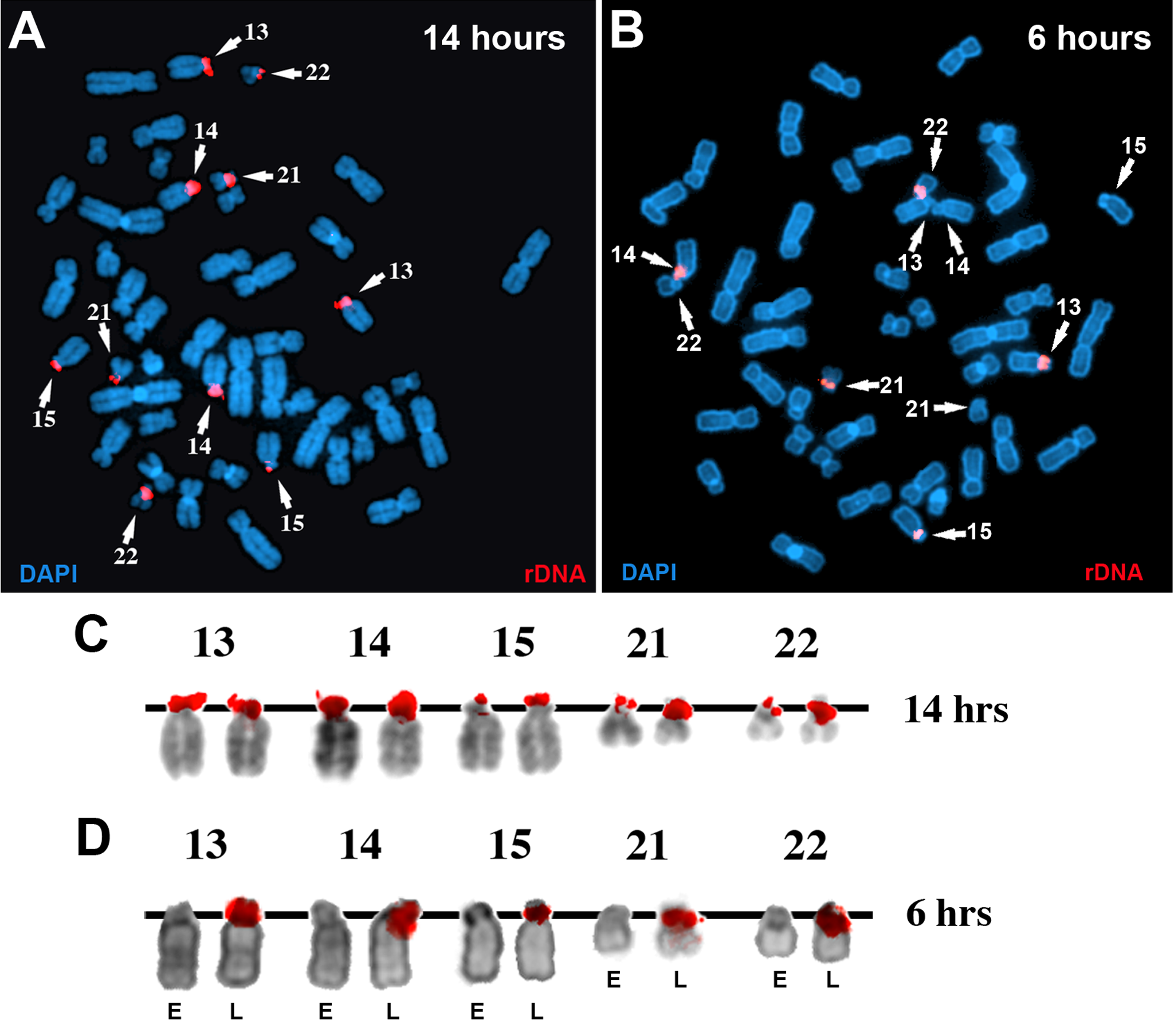

Supplement: Figure S1 — ReTiSH assay on rDNA loci in primary blood lymphocytes. Human lymphocytes were obtained from normal individuals and cultured for less than one week. Cells were labeled with BrdU for 14 or 6 hours, arrested in metaphase, and subjected to ReTiSH using an rDNA probe (red). The replicated rDNA alleles were detected using a PCR fragment representing 18S rDNA, and the DNA was detected with DAPI. Arrows mark the 10 chromosomes containing rDNA clusters. A) A single metaphase spread from the 14 hour time point. B) A single metaphase spread from the 6 hour time point. C and D) The DAPI images from the 10 rDNA containing chromosomes from panels A and B were inverted and the banding patterns were used to identify the 5 rDNA containing chromosomes. C) The ReTiSH signals from a representative cell (panel A) harvested at the 14 hour time point and hybridized to the rDNA probe (red) are shown. D) The ReTiSH signals from a representative cell (panel B) harvested at the 6 hour time point and hybridized to the rDNA probe (red) are shown. The early (E) and late (L) replicating chromosomes for each pair of homologs are indicated. (TIF) [file pgen.1003423.s001.tif]

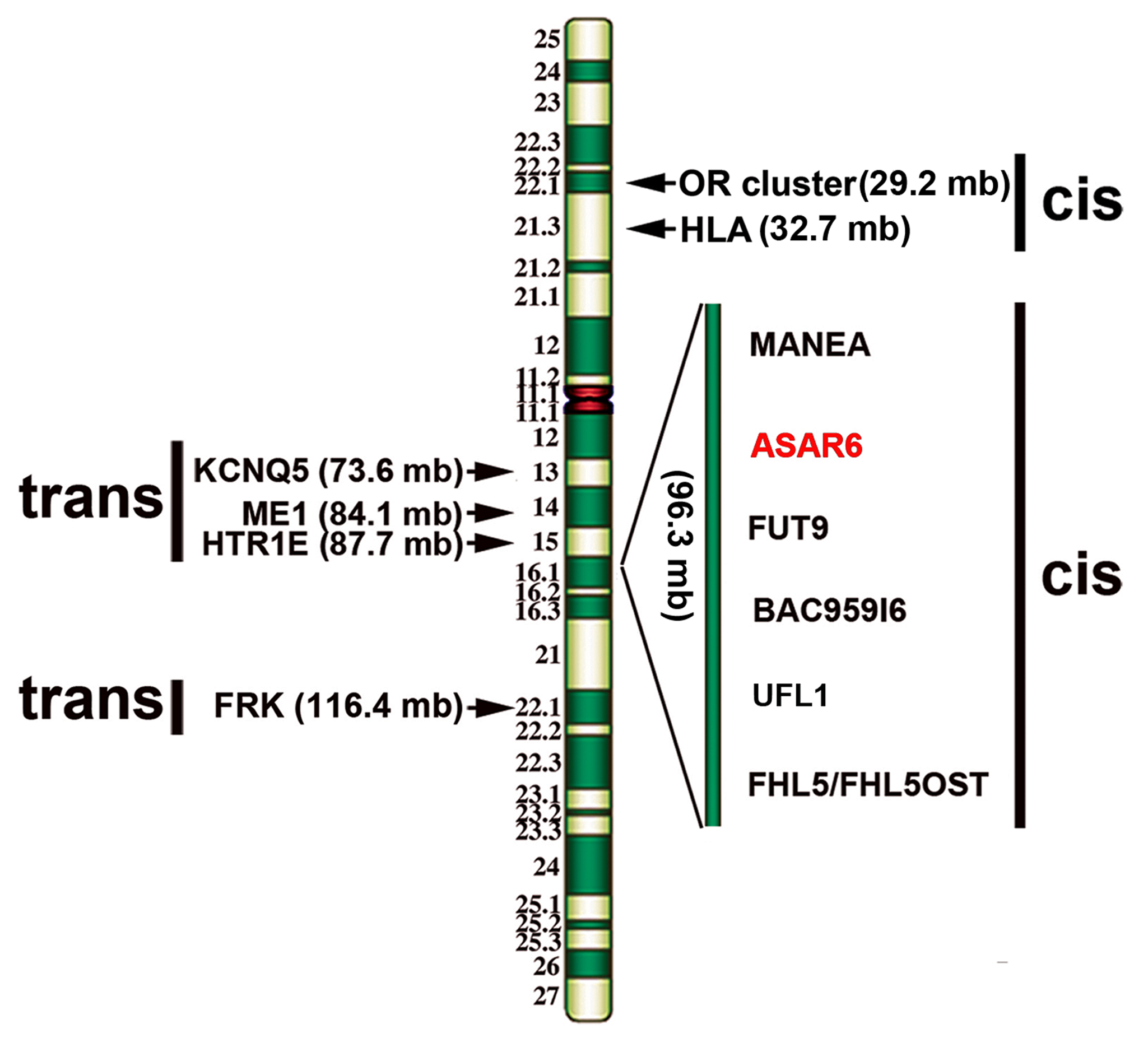

Supplement: Figure S2 — Schematic diagram of chromosome 6 showing the location of the genes and loci assayed for asynchronous replication. The ∼1.2 mb region of chromosome 6 between MANEA and FHL5/FHL5OST is expanded on the right. The coordination in asynchronous replication of chromosome 6 mono-allelically expressed genes with ASAR6 was found to be either in cis or in trans. (TIF) [file pgen.1003423.s002.tif]

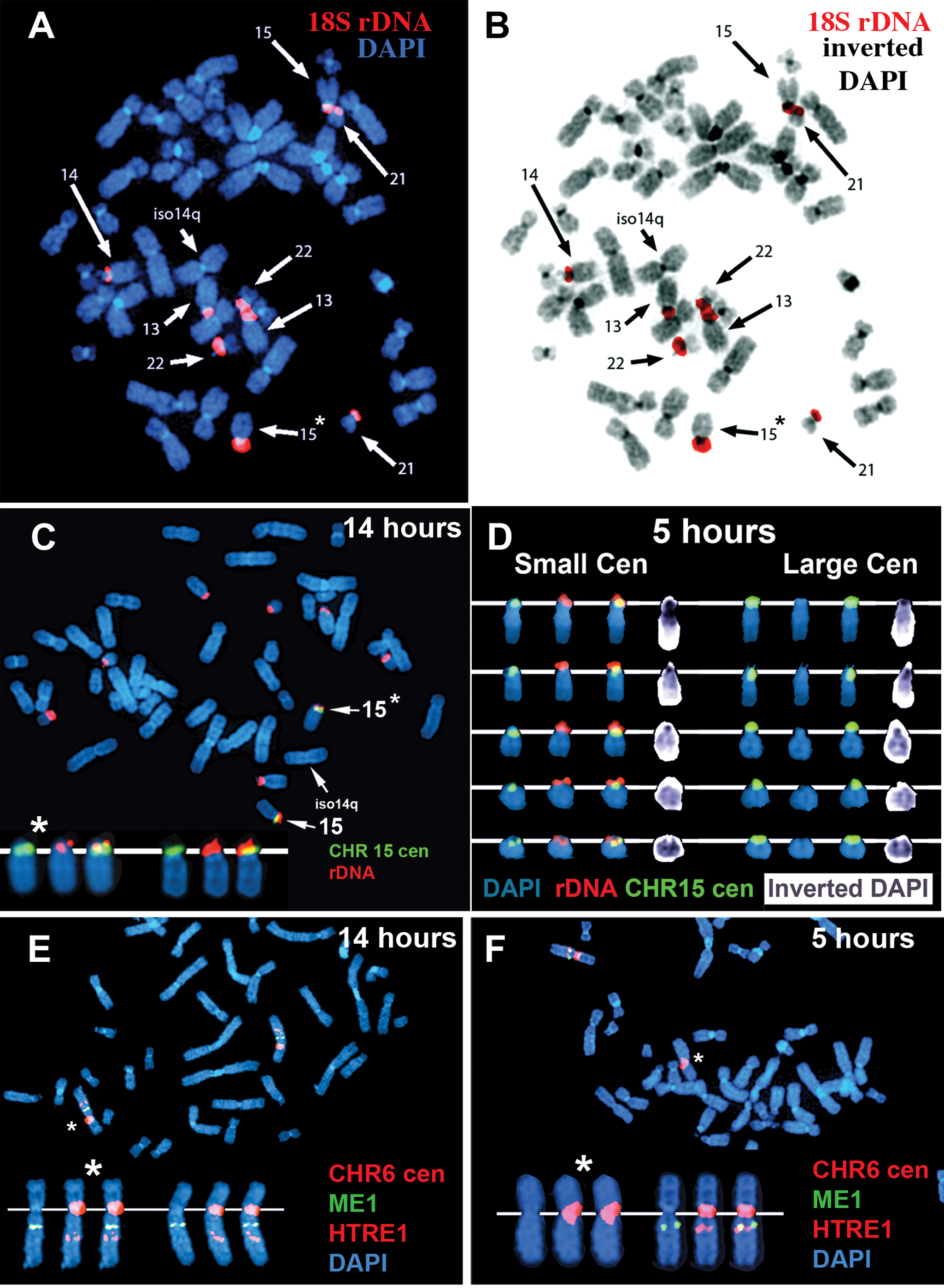

Supplement: Figure S3 — ReTiSH assay in P175 cells. A) Conventional DNA FISH on P175 cells using the rDNA as probe. Note that P175 cells contain 9 chromosomes that contain rDNA clusters. This is because one of the chromosome 14 s became an isochromosome [iso(14q)] and during this process a deletion of the rDNA cluster occurred. Arrows mark the chromosomes containing rDNA clusters plus the iso(14q). B) Inverted DAPI staining of the mitotic cell shown in panel A. The inverted DAPI banding pattern was used to identify chromosomes 13, 14, 15, 21, and 22. The asterisk marks the chromosome 15 containing the larger centromere polymorphism. C). 14 hour ReTiSH on P175 cells using the rDNA probe plus a chromosome 15 centromeric probe. The asterisk marks the chromosome with the larger centromeric signal. D) 5 hour ReTiSH on five representative cells probed with the rDNA (red) and chromosome 15 centromeric (green) probes. The chromosome 15 s were cut out from each cell and aligned with their centromeres on a white line. The chromosome 15 s containing the small and large centromeric polymorphism from 5 representative cells are shown. E and F) ReTiSH assay on P175 cells probed with HTRE1 (red), ME1 (green), and a chromosome 6 centromere (red). The asterisk marks the chromosome 6 with the larger centromere. (TIF) [file pgen.1003423.s003.tif]

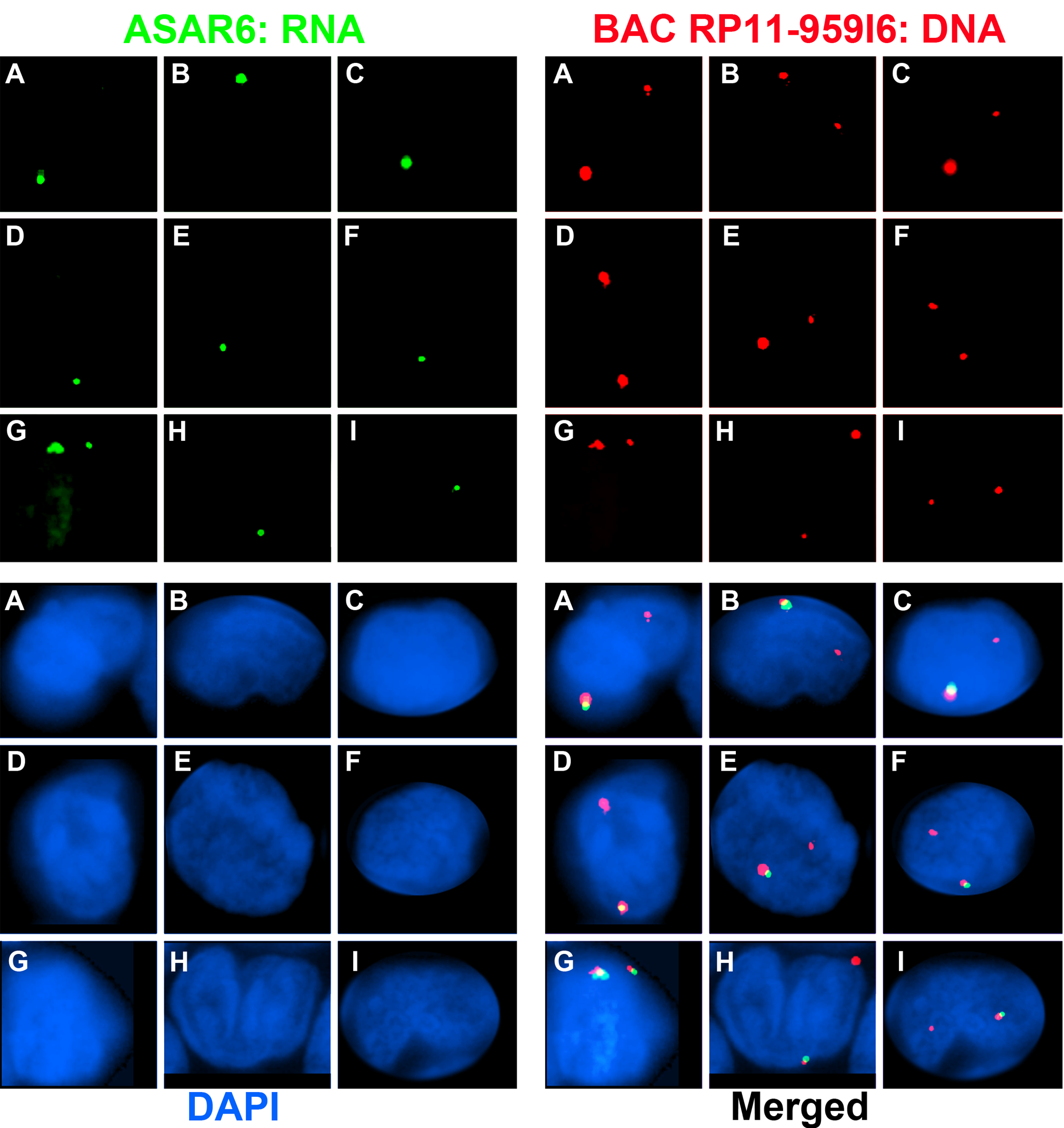

Supplement: Figure S4 — RNA-DNA FISH for expression of ASAR6. PBLs were subjected to RNA FISH (green) using a Fosmid (G248P86031A6) probe for ASAR6. Slides were subsequently re-fixed and processed for DNA FISH (red) using BAC RP11-959I6, located distal to FUT9 (BAC#4 in Figure 1H). The four sets of panels (A–I) show the same cells used in Figure 4A–4I, except that each color is displayed separately or merged (bottom right). (TIF) [file pgen.1003423.s004.tif]

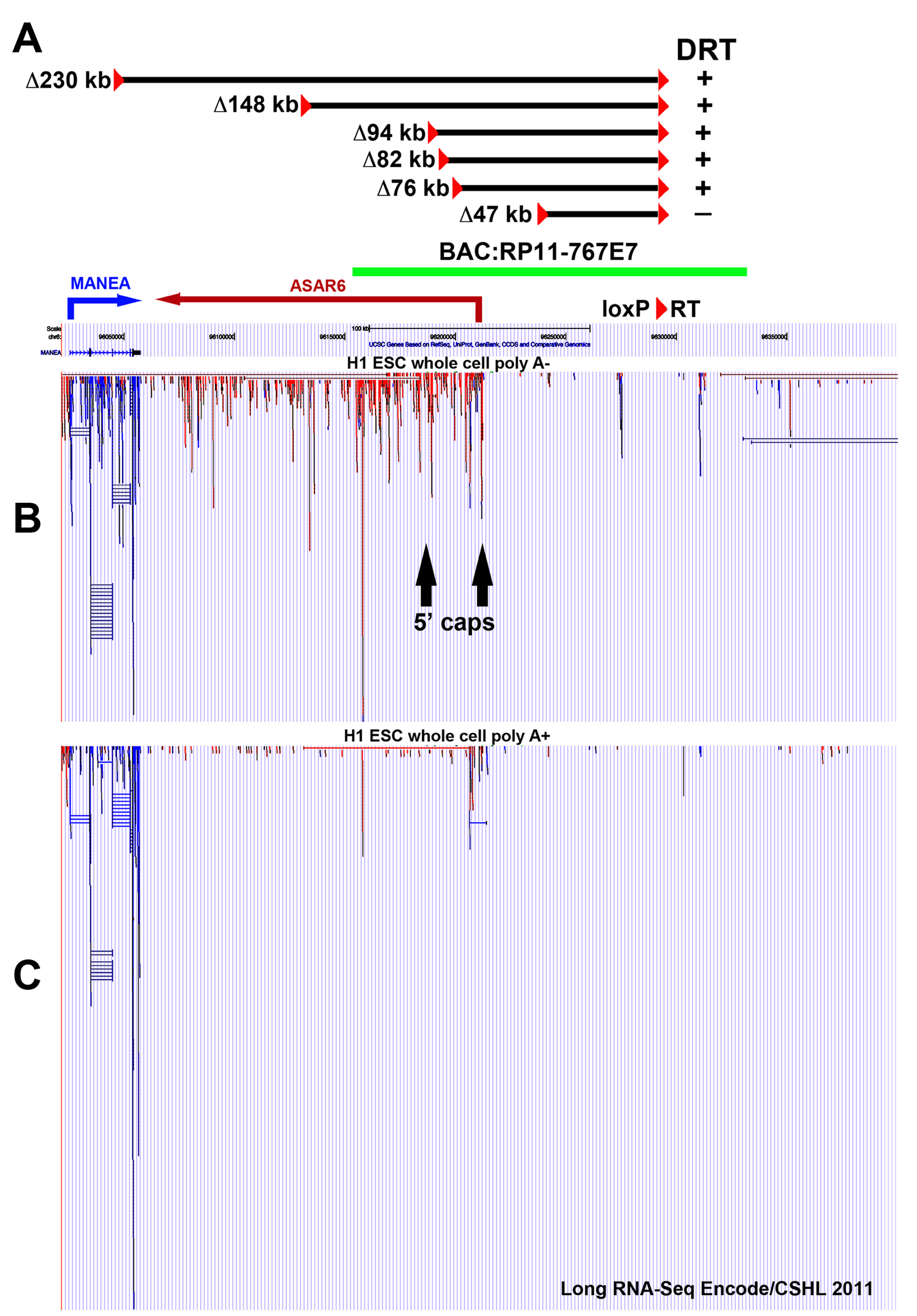

Supplement: Figure S5 — Schematic illustration of the ASAR6 locus. The locations of MANEA, ASAR6, BAC RP11-767E7, the original loxP integration site [loxP(red triangle)RT] and 6 different deletions in P175 cells [9] are depicted above a screenshot of the UCSC Genome Browser of this region of chromosome 6. A) A set of nested deletions was generated in P175 cells, all except the smallest ∼47 kb deletion (Δ47) display DRT. B and C) UCSC Genome Browser view of the RNA-seq data from whole cell poly A− (B) or poly A+ (C) RNA from the human ES cell line H1 [25]. The blue tick marks indicate sequence hits from the + direction, and the red tick marks indicate sequence hits from the - direction. Note that ASAR6 RNA is enriched in the poly A− fraction, while MANEA RNA is detected in both poly A− and poly A+ fractions. The locations of 5′ caps from the Encode/RIKEN CAGE [63] track are also shown. (TIF) [file pgen.1003423.s005.tif]

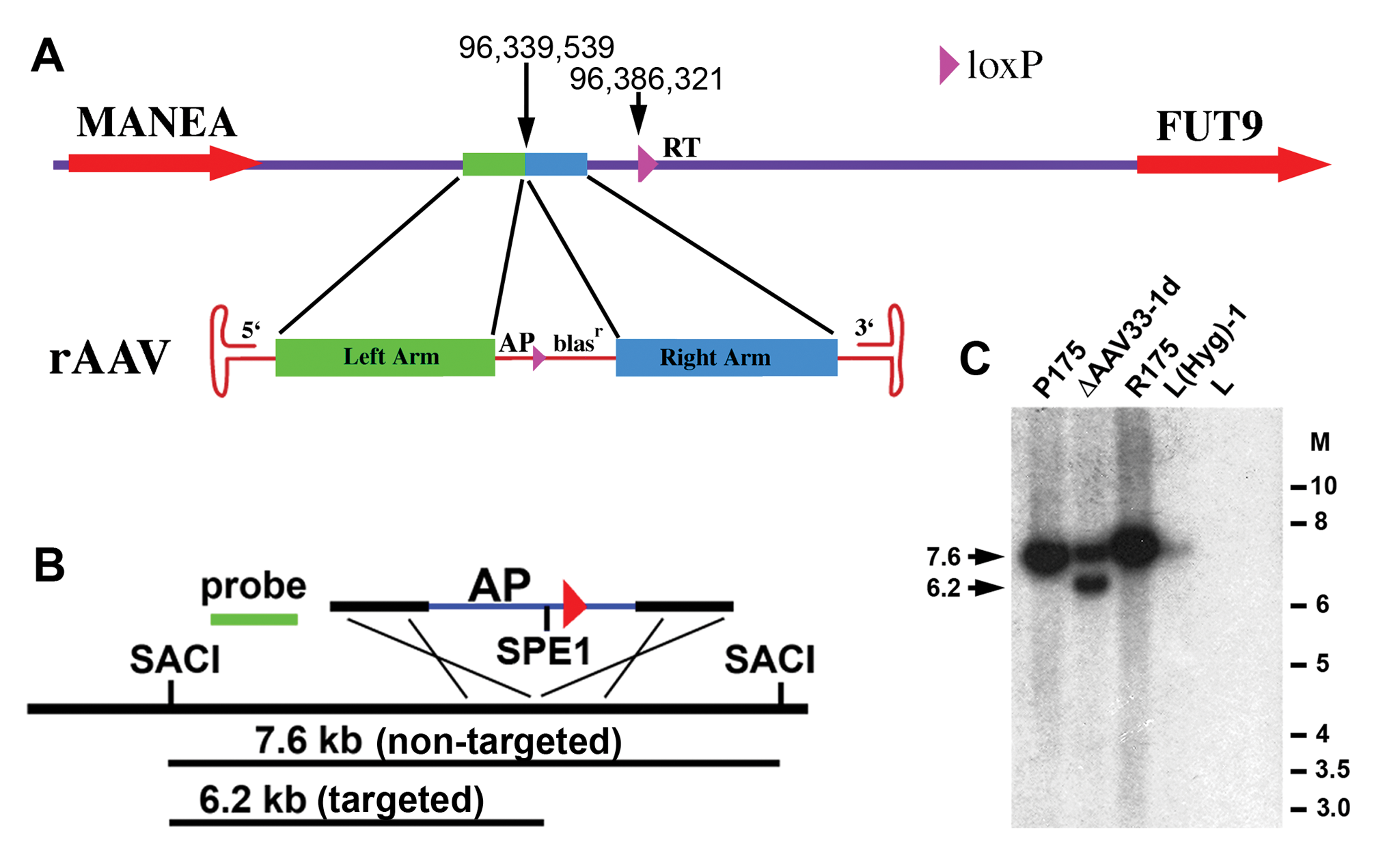

Supplement: Figure S6 — rAAV strategy for generating the ∼47 kb deletion upstream of ASAR6. A) Left and right arms of homology upstream of ASAR6 were cloned into the pAAV-MCS vector (Stratagene). In addition, a loxP cassette containing the 5′ portion of the APRT gene (AP) plus the blasticidin resistance gene (blasr) are shown. B) Southern blot hybridization scheme including the location of the probe, which is outside of the homology arms used for targeting, is shown. C) Southern blot hybridization illustrating correct integration of the loxP cassette is shown. Genomic DNAs were digested with SAC1 and SPE1. Note that the loxP cassette inserts a SPE1 site into the targeted locus. Control DNAs included the parental P175, R175 [containing a t(6;10) at the original loxP site in P175 cells] and a mouse L cell somatic cell hybrid containing human chromosome 6. (TIF) [file pgen.1003423.s006.tif]

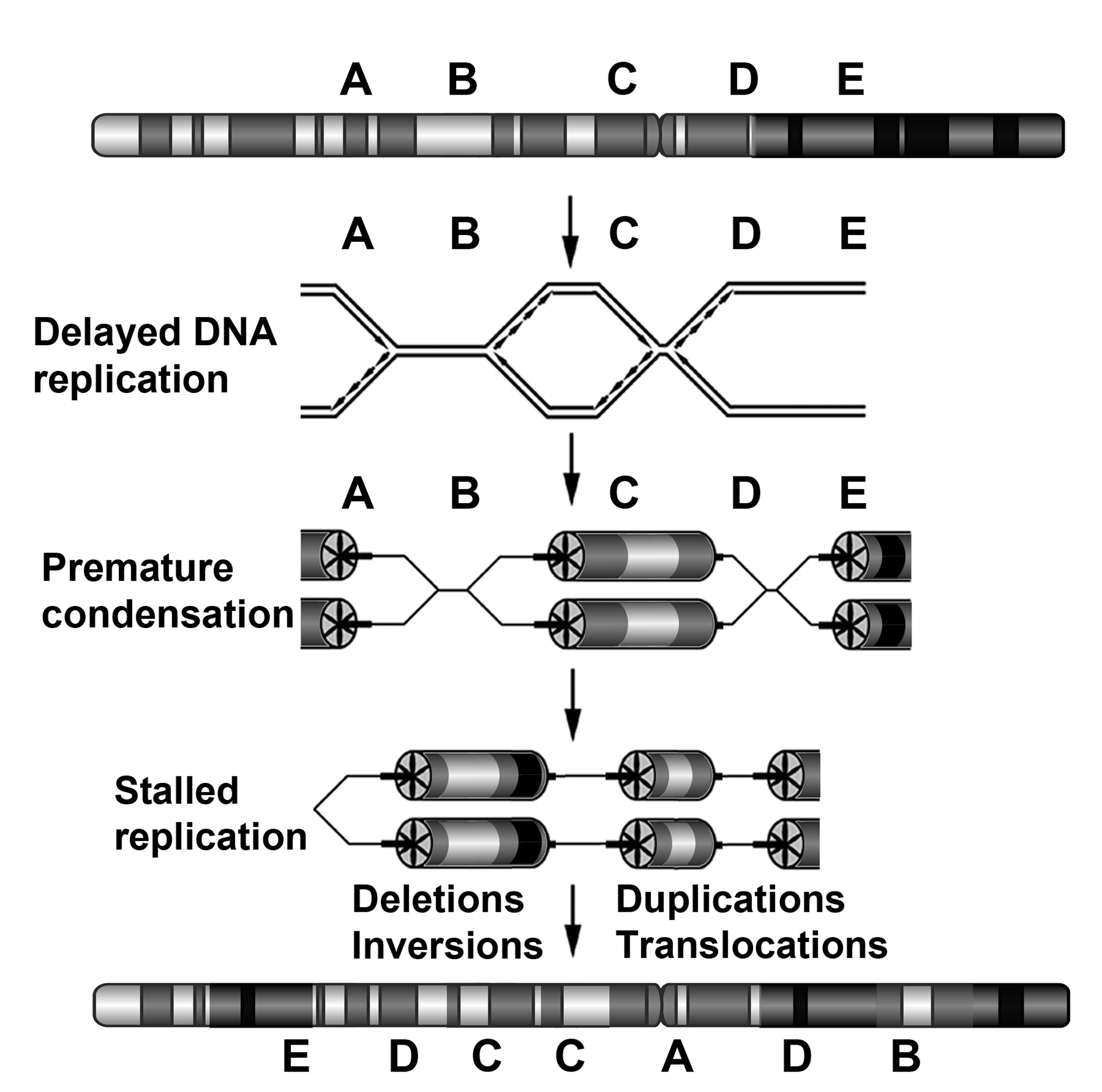

Supplement: Figure S7 — Model for structural instability of individual chromosomes. Disruption of an inactivation/stability center leads to delayed replication timing of an individual chromosome. A human chromosome is depicted as a banded cylinder, and the original order of loci along the chromosome are indicated by the letters A–E. Delayed replication timing leads to delayed mitotic chromosome condensation and the onset of mitotic chromosome condensation prior to the completion of DNA synthesis (Premature condensation). This Premature condensation leads to stalled replication forks, which are depicted as X and Y structures. Multiple rearrangements (deletions, inversions, duplications, and translocations) are subsequently generated at the stalled forks via replicative mechanisms. The new order of loci are indicated with the letters E-B. (TIF) [file pgen.1003423.s007.tif]
